# Optical detection of magnetic resonance

**DOI:** 10.5194/mr-1-115-2020

**Published:** 2020-06-30

**Authors:** Dieter Suter

**Affiliations:** Experimental Physics III, TU Dortmund University, 44227 Dortmund, Germany

## Abstract

The combination of magnetic resonance with laser spectroscopy provides some interesting options for increasing the sensitivity and information content of magnetic resonance. This review covers the basic physics behind the relevant processes, such as angular momentum conservation during absorption and emission. This can be used to enhance the polarization of the spin system by orders of magnitude compared to thermal polarization as well as for detection with sensitivities down to the level of individual spins. These fundamental principles have been used in many different fields. This review summarizes some of the examples in different physical systems, including atomic and molecular systems, dielectric solids composed of rare earth, and transition metal ions and semiconductors.This review was originally written in response to an invitation of “Progress in NMR Spectroscopy” but re-directed to *Magnetic Resonance* to be accessible to a wide audience. This paper has been reviewed by peers in accordance with the policy of *Magnetic Resonance*.

This review was originally written in response to an invitation of “Progress in NMR Spectroscopy” but re-directed to *Magnetic Resonance* to be accessible to a wide audience. This paper has been reviewed by peers in accordance with the policy of *Magnetic Resonance*.

## 1 Introduction and overview

### 1.1 Sensitivity of magnetic resonance

Magnetic resonance spectroscopy basically measures the interaction of electronic or nuclear angular momenta with each other and with external magnetic fields [Bibr bib1.bibx1]. While the fundamental processes that occur during magnetic resonance have been understood for at least 70 years, the field has continued to expand in many directions, mostly due to the ever increasing possibilities of using spins as probes of their environment. Today, the biggest remaining weakness of the technique is its relatively low sensitivity, compared, for example, to optical experiments. In the area where magnetic resonance spectroscopy has become most popular, that of nuclear magnetic resonance (NMR) of liquids, the minimum number of spins that can be detected, is of the order of $10^{17}$ . In contrast to this, the ultimate sensitivity limit, i.e. spectroscopy of individual particles, was demonstrated for optical systems 4 decades ago [Bibr bib1.bibx73].

Several issues contribute to this low sensitivity. The main reason is that the interaction energies are relatively small ( $<10^{-22}$ J), so that the corresponding frequencies are in the radio-frequency (RF) or microwave (MW) regime ( $<10^{11}$ Hz). The small interaction energy results e.g. in small thermal population differences between the energy levels participating in a particular transition, which are determined by the Boltzmann factor $e^{-h\nu /k_{B}T}$ . For optical transitions, where ν is of the order $10^{18}$ Hz, the population is almost completely confined to the ground state, as shown graphically in Fig. [Fig Ch1.F1]. For RF transitions ( $\nu <10^{9}$ Hz), however, the populations are almost identical, with differences $<10^{-4}$ . Similarly, the energy of an RF photon is, under typical conditions, well below the thermal noise level. This makes it very difficult to detect a single RF photon, in contrast to optical photons, which can be detected with efficiencies close to unity.

**Figure 1 Ch1.F1:**
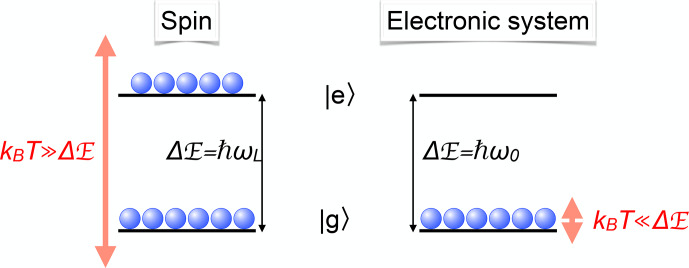
Comparison of spin and optical transitions with the corresponding populations at room temperature.

### 1.2 Optics and lasers

While magnetic resonance is concerned with transitions between states that differ in terms of their spin quantum numbers, optical transitions occur between states that differ in terms of their electronic configuration. The energy difference between these states is typically of the order of electron volts (eV), which corresponds to transition frequencies of $\approx 4\times 10^{14}$ Hz. Since this energy difference is large compared to the thermal energy of the system, only the lowest states are populated in thermal equilibrium, as shown in Fig. [Fig Ch1.F1].

Early experiments on optical excitation and detection of magnetic resonance used conventional light sources such as discharge lamps. These light sources had a very limited intensity, which implied that the systems with which they interacted were only weakly perturbed. A significant effect, e.g. in terms of establishing a significant population difference between the spin states, could only be achieved if the relaxation processes could be kept to a minimum. In those early experiments, light was used mainly in order to polarize the spin system and to observe the precessing magnetization, while RF irradiation was used to change the dynamics of the spin system. Nevertheless, it was realized early [Bibr bib1.bibx22] that optical radiation cannot only polarize the spin system, but also leads to shifts and broadening of the magnetic resonance transitions. With the introduction of the laser, the available light intensity and the coherence properties of the radiation field changed in such a way that many experiments that had not been feasible before have become routine [Bibr bib1.bibx26]. One important example is the generation of ultrashort laser pulses, which provide high intensity as well as high time resolution.

The advent of the laser also led to the revisions on the theoretical side. In particular, the high spectral purity and large intensity of the laser light result in a nonlinear response of the system to the optical field and to additional phenomena such as selective excitation. In many cases, the optical coherences have to be taken into account, and the dynamics must be formulated in terms of the density operator [Bibr bib1.bibx25]. Other effects, which were discovered with discharge lamps but were too small to be of practical significance, were increased by many orders of magnitude. For example, the light-shift effect, an apparent shift of energy levels due to optical irradiation of an adjacent transition, has the same effect on the spin dynamics as magnetic fields. By selectively irradiating certain optical transitions, these virtual magnetic fields can be used as an additional degree of freedom for the modification of spin dynamics. It is therefore possible to perform many experiments by purely optical methods; the usage of the optical radiation field is then 3-fold: it polarizes the spin system by transferring angular momentum from the photons to the spin system, it modifies the dynamics of the system via an effective Hamiltonian, and it is used to detect the resulting time-dependent magnetization.

### 1.3 Coupled optical and magnetic resonance transitions

While the systems under study can have very different energy level schemes, the basics of the techniques can often be explained in terms of a simple three-level scheme (Fig. [Fig Ch1.F2]). The transition of interest is between the two spin substates of a given electronic state, i.e. between the two ground states in the case of the Λ -type system and between the two excited states in the V -type system (right-hand part of Fig. [Fig Ch1.F2]). In many actual cases, both types of transitions occur in the same system, so that resonances in both the ground and excited states can be excited and detected.

**Figure 2 Ch1.F2:**
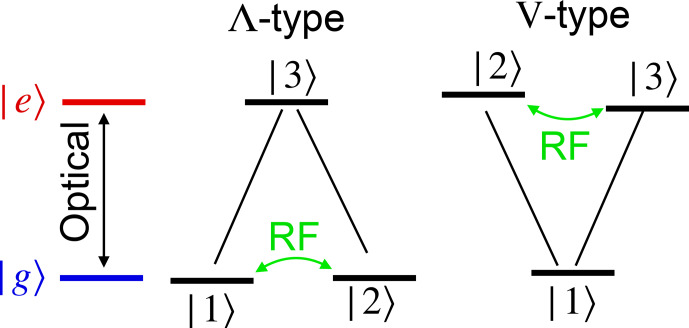
The most important three-level systems that allow basic optical excitation and detection of magnetic resonance. The arrows indicate the transitions coupling to optical and RF photons.

### 1.4 Optical pumping and optical detection

In cases where the sensitivity provided by classic magnetic resonance is not sufficient, it is often possible to increase the population difference between the different magnetic sublevels by optical pumping (see e.g. [Bibr bib1.bibx7]). Like the population difference between ground and electronically excited states, the population difference between levels differing only in their spin state can then reach values near unity.

Conversely, the population difference and coherence between the magnetic substates can change the optical properties of the system; it is therefore possible to detect the magnetization optically, with a sensitivity much greater than if the radio frequency photons are detected [Bibr bib1.bibx51]. In simple cases, this gain in sensitivity can be understood as an amplification of the radiation by transferring the angular momenta from the internal degrees of freedom of the system to photons with optical energies instead of RF energies. In classical terms, this transfer of angular momentum basically leads to a (circularly) polarized radiation field.

### 1.5 Motivation

Optical techniques have turned out to be useful in many different areas of magnetic resonance. As pointed out above, the main motivation is often the gain in sensitivity, which also has allowed us to reach the single-spin limit [Bibr bib1.bibx108]. Apart from the gain in sensitivity, the use of optical radiation also provides the option of performing magnetic resonance spectroscopy of electronically excited states. Since these states are not populated in thermal equilibrium, the targeted systems must be brought into the excited state before magnetic resonance can be performed. If the excitation can be achieved with light, it is often advantageous to use selective excitation of the magnetic substates to obtain a spin-polarized system. This is also necessary since the excited state population that can be achieved may be substantially smaller than in the ground state, so that sensitivity again becomes an important issue. The fluorescence emitted by these systems is often polarized and can be used directly to measure the excited state magnetization.

A third reason to combine magnetic resonance with optical techniques is that the information content of double-resonance experiments is often higher than the information that can be obtained with the individual techniques. This includes e.g. the identification of fluorescent centres through their magnetic resonance spectra and selective excitation and detection of magnetic resonance near a surface [Bibr bib1.bibx40] or in selected semiconductor quantum wells [Bibr bib1.bibx31]. In some cases, the double-resonance experiment allows one to break symmetries inherent in magnetic resonance. Breaking them in a controlled way can allow one to differentiate between positive and negative signs in some coupling constants [Bibr bib1.bibx75] or to obtain orientational information from an isotropic medium such as a frozen solution [Bibr bib1.bibx18].

## 2 Physical background

Combining optical methods with magnetic resonance is possible if a system has electronic as well as spin degrees of freedom. Optical fields generally interact with transitions between different electronic states through the electric dipole interaction, while magnetic resonance drives transitions between states that differ with respect to their spin (or, more generally, angular momentum) degrees of freedom. The two subsystems are often coupled, since the optical transitions connect states that differ not only with respect to their electronic configuration, but also in terms of their angular momentum.

### 2.1 Angular momentum and selection rules

Spins are an important form of angular momentum and magnetic resonance is the main approach to excite and detect transitions between states that differ only in angular momentum. In the case of nuclear magnetic resonance, the spin angular momentum of atomic nuclei is the object under investigation, while in the case of EPR, the spin of the electron can be mixed with orbital angular momentum of the electron. Radiation also carries angular momentum. While it is possible to determine the angular momentum of an electromagnetic wave in terms of Maxwell's equations, it becomes much more relevant in quantum mechanics, where each photon carries a spin angular momentum of ℏ . In vacuum, it is sufficient to consider two of the three angular momentum states, e.g. the cases where the spin is oriented parallel to the direction of propagation, and opposite to it. These states correspond to circularly polarized light.

If the environment of the object being studied is isotropic, such as in a free atom, angular momentum is a conserved quantity. Accordingly, a change in the spin state must be compensated by a change in some other form of angular momentum. This is important e.g. during absorption or emission of photons: the angular momentum of the photon that is created or destroyed must be compensated by a corresponding change in the angular momentum state of the system that absorbs (or emits) the photon.

Figure [Fig Ch1.F3] shows this exchange of angular momentum between matter and radiation field for a simple case taken from atomic physics. In this example, the electronic ground state as well as the electronically excited states have an electronic angular momentum of J=ℏ/2 . This corresponds e.g. to the $D_{1}$ line of alkali atoms, where the ground-state angular momentum is given by the spin S=ℏ/2 of the electron, while its orbital angular momentum vanishes. The first excited state has an orbital angular momentum of L=ℏ and is split into the J=L±S states ${}^{2}P_{3/2}$ and ${}^{2}P_{1/2}$ , which are connected to the ground state by the $D_{2}$ and $D_{1}$ lines. If the atom absorbs a photon, it must change its internal state such that the angular momentum $J^{\prime }$ of the final state of the atom is equal to the sum of the initial angular momentum J of the atom plus the spin angular momentum s of the photon, $J^{\prime }=J+s$ .

**Figure 3 Ch1.F3:**
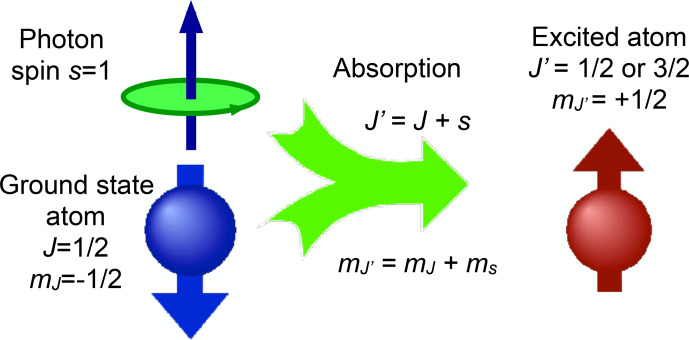
A simple absorption process illustrating angular momentum conservation in atomic physics.

### 2.2 Optical pumping

Exciting spins in magnetic resonance requires that the states do not all have the same occupation probability. In conventional magnetic resonance experiments, thermal contact of the spins with the lattice establishes the polarization. This process is relatively slow, especially at low temperatures where relaxation times can be many hours. In addition, the polarization is limited by the Boltzmann factor, which is typically less than $10^{-4}$ . Much higher polarizations can be achieved by transferring population differences from photons, which can easily be prepared in pure states, i.e. with a polarization of 100 %. This transfer process is known as optical pumping [Bibr bib1.bibx51].

Figure [Fig Ch1.F4] illustrates the basic process of optical pumping for a simple four-level system. In this example, the electronic ground state as well as the electronically excited state have total angular momentum J=1/2 . If the laser field is circularly polarized, it contains only photons whose angular momentum $J_{p}=1$ (in units of ℏ ) is oriented parallel to the direction of propagation. The system that absorbs the photon has to accommodate its energy as well as its angular momentum. The energy is absorbed by changing from the electronic ground state to the electronically excited state, while the angular momentum is absorbed by changing from the $m_{J}=-1/2$ to +1/2 states.

**Figure 4 Ch1.F4:**
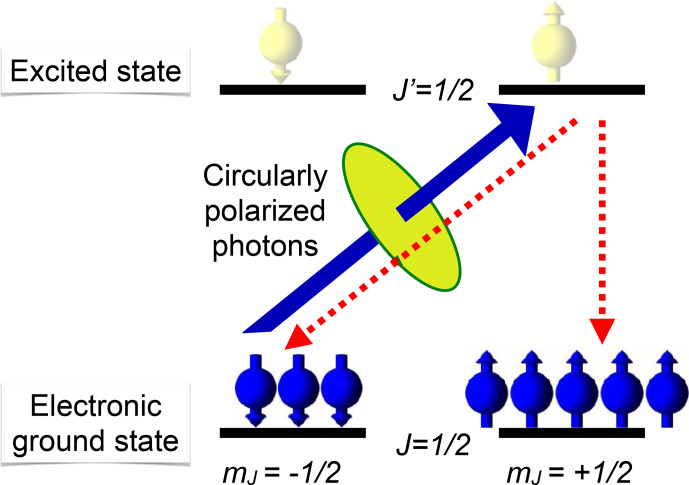
Basic principle of optical pumping. The solid arrow indicates the only transition that couples to a circularly polarized laser field in this level system. The dashed arrows indicate transitions for spontaneous emission.

Since the excited state is not stable, the system falls back into the ground state. During this process, the absorbed photon energy is dissipated either as a photon (radiative relaxation) or by transfer to other degrees of freedom (radiationless). In both cases, the system can end up in either of the two ground states. Since the |g,↑〉 state does not couple to the laser field, the population of this state grows continuously, if the process is repeated, and the system can be pumped into this state.

While this simplified level scheme is quite useful for understanding the basic processes occurring during optical pumping, it is important to consider real systems for any quantitative analysis. In particular, the presence of a nuclear spin has very significant effects, apart from the existence of hyperfine splitting. As an example, the optical pumping process in the true atomic ground state is nonexponential and slower by at least an order of magnitude, compared to a hypothetical atom with a vanishing nuclear spin [Bibr bib1.bibx94].

### 2.3 Other mechanisms for optical polarization

The basic mechanism for optical pumping described above is easily understood in terms of angular momentum conservation. In solid-state systems, however, space is not isotropic and angular momentum is in general not a preserved quantity. In such systems, it is sometimes possible to obtain polarization of electronic as well as nuclear spins via different optical pumping processes.

An important example is the case of spin-selective intersystem crossing (ISC), where electronic singlet states convert to triplet states and vice versa. Figure [Fig Ch1.F5] shows the basic principle for the two most important cases. On the left-hand side, the ground state is a singlet state. A laser photon brings it to an excited singlet state, which has a finite probability of making a radiationless transition to a triplet state. The interactions between the unpaired electron spins in the triplet state and their spin–orbit interaction give rise to the zero-field splitting which completely or partially lifts the degeneracy of the spin states. The ISC transition rates from the singlet to different triplet states depend on the spin–orbit coupling and can therefore differ significantly, resulting in different populations of the triplet states. Similarly, the triplet state lifetimes are in general different, as indicated by the different thickness of the arrows that connect the triplet states to the singlet ground state. These different lifetimes again lead to unequal populations of the three triplet states.

**Figure 5 Ch1.F5:**
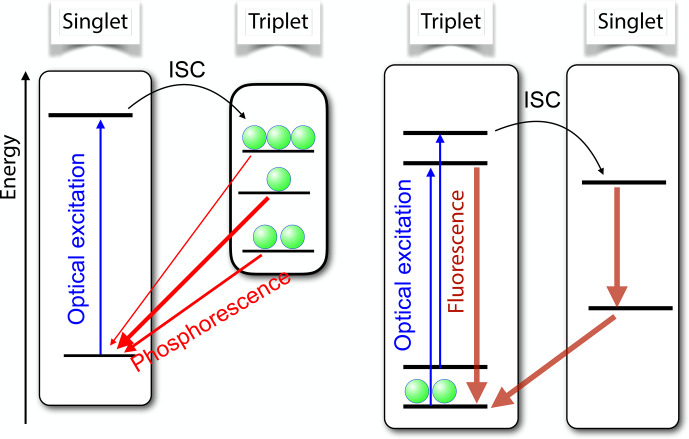
Basic principle of spin-selective intersystem crossing (ISC) for the case of a singlet ground state (left) and a triplet ground state (right). In both cases, the optical irradiation results in unequal populations of the triplet states.

If a resonant microwave field drives one of the transitions between the triplet states, this affects the populations and therefore also the photon emission rates. Accordingly, this system is quite well suited for measuring the energy differences between excited triplet levels via optical detection. In addition, the polarization of the electron spin can be transferred to coupled nuclear spins. The resulting nuclear spin polarization survives the transition into the singlet ground state, where it can accumulate over multiple absorption–emission cycles.

The right-hand part of Fig. [Fig Ch1.F5] shows a different type of system, where the ground state is a triplet state. Absorption of laser photons and direct emission of photons in a zero magnetic field is in general spin-preserving. However, the different spin states can have vastly different probabilities of undergoing ISC to the singlet state. In the important case of the nitrogen-vacancy (NV) centre in diamond, this allows one to pump most of the electron spin population into the $m_{S}=0$ state of the electronic ground state [Bibr bib1.bibx27].

Examples where this mechanism generates high spin polarization include organic molecules like pentacene, where the first sign of high electron polarization was suggested by [Bibr bib1.bibx103] and precise determination of polarization by transient ESR was reported by [Bibr bib1.bibx92]. More recently, the transfer of polarization to nuclear spins was reported [Bibr bib1.bibx58]. Other systems include quinoxaline [Bibr bib1.bibx104], oxygen-vacancy complexes in silicon [Bibr bib1.bibx49], and the NV centre in diamond [Bibr bib1.bibx27], which is discussed in detail in Sect. [Sec Ch1.S4.SS3]. Similar processes are responsible for the polarization of spins in quartet states that undergo ISC to doublet states, such as some defects in SiC [Bibr bib1.bibx9].

### 2.4 Optical detection

Classical magnetic resonance relies mostly on the detection of time-dependent magnetization by coupling an oscillating component of the associated magnetic flux to an external antenna like a coil or a microwave cavity. In most cases, this coupling can be well described by Faraday's law of induction or, equivalently, Maxwell's third law. The optical detection techniques that are discussed in this section, however, do not depend on magnetic flux. Instead, the angular momentum (mostly spin) couples directly or indirectly to the angular momentum of some optical photons or to other degrees of freedom of the optical field. The possibility of using optical properties for detecting magnetic resonance was first suggested by [Bibr bib1.bibx14].

In the following, we start with a relatively general discussion, where the spins can be either electronic or nuclear spins, and they may be located in solid or gaseous samples (liquids are less suitable for this type of experiment). We will therefore refer to them with the general term “particles”, which will stand for all types of spin-carrying centres under study.

#### 2.4.1 Absorption/transmission

Figure [Fig Ch1.F6] illustrates a simple mechanism for optical detection: light with a given circular polarization interacts only with one of the transitions between the ground state |g〉 and the excited state |e〉 . We assume for simplicity that all particles are in the electronic ground state, but the two spin states have different populations. Since the absorption of the medium is directly proportional to the number of atoms that interact with the light, a system with spin polarization is circularly birefringent and dichroic; i.e. the refractive index and absorption coefficients for the two opposite circular polarizations are different. Comparison of the absorption or dispersion of the medium for the two opposite circular polarizations yields a signal that is proportional to the population difference and thus to the spin polarization along the direction of propagation. This type of measurement is often used e.g. in different forms of experiments in an ultra-low magnetic field. Some examples are discussed in Sect. [Sec Ch1.S3.SS1].

**Figure 6 Ch1.F6:**
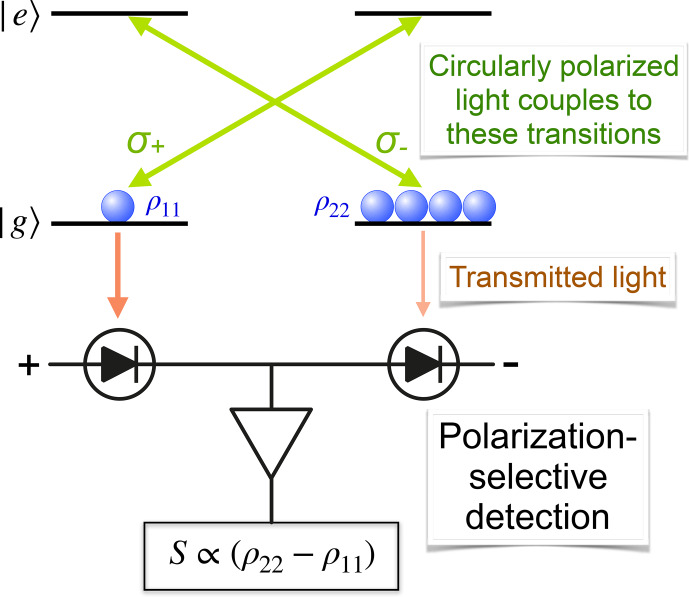
Basic principle of optically detecting magnetic resonance in transmission by differential absorption.

In the ideal situation, where a particle in a spin state |↑〉 ( |↓〉 ) absorbs only left (right) circularly polarized light, a beam of light that has initially the same number $n_{p_{\pm }}(0)$ of photons for both circular polarizations undergoes differential absorption. Behind the sample of length ℓ , the numbers are

$$n_{p_{\pm }}(\ell )=n_{p_{\pm }}(0)e^{-\ell \alpha_{0}N_{0}p_{\pm }},$$

where $\alpha_{0}$ is the absorption coefficient per particle, $N_{0}$ the total particle density, and $p_{\pm }$ the fraction of spins in the states ↑ / ↓ . If the exponent is small, we can use a linear expansion

$$n_{p_{\pm }}(\ell )=n_{p_{\pm }}(0)\left(1-\ell \alpha_{0}N_{0}p_{\pm }\right).$$

Difference detection, as shown in Fig. [Fig Ch1.F6], for initially equal photon numbers, $n_{p_{+}}(0)=n_{p_{-}}(0)=n_{0}$ , yields

1 $$\Delta n(\ell )=-2n_{0}\ell \alpha_{0}N_{0}\left(p_{+}-p_{-}\right)=-2n_{0}\ell \alpha_{0}N_{0}\Delta p,$$

where we have used the fractional spin polarization $\Delta p=p_{+}-p_{-}$ . Since difference detection is free of background, this signal is not significantly perturbed by classical noise of the laser. It is, however, affected by shot noise, which was not considered in this classical analysis. According to Eq. ([Disp-formula Ch1.E1]), a high sensitivity (i.e. small $N_{0}$ and Δp ) can be achieved by using a large $n_{0}$ (i.e. high laser intensity), a long path length ℓ , and a large absorption coefficient $\alpha_{0}$ . These goals tend to be incompatible, however. As an example, large absorption coefficients and long path lengths lead to an inhomogeneous system and violate the assumption of linearity made in this derivation, while the combination of high laser intensity and large absorption coefficient leads to unwanted perturbations of the system. In cases where these issues become important, it is possible to modify the basic scheme discussed above, e.g. by using dispersive instead of absorptive detection [Bibr bib1.bibx98]. In this case, the complex index of refraction (i.e. absorption as well as dispersion) depends linearly on the spin polarization of the ground state [Bibr bib1.bibx84].

#### 2.4.2 Spontaneous emission

When electronically excited states are populated during an experiment, their return to the ground state may be accompanied by the emission of a photon that carries information about the state that was populated. While it is much harder to detect spontaneously scattered photons, since they are emitted over a large solid angle, they provide a significantly higher information content than the transmitted laser photons: they are all emitted (if correctly filtered) by the system under study.

**Figure 7 Ch1.F7:**
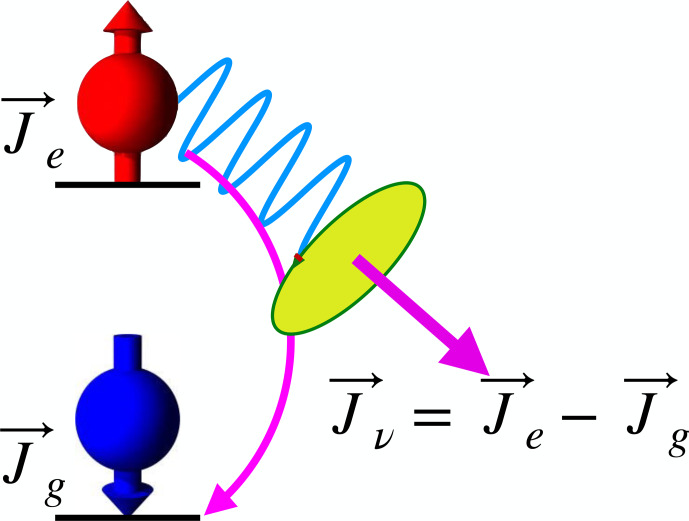
Angular momentum conservation during photon emission.

Whether these photons are actually useful depends on the system. Figure [Fig Ch1.F7] shows a simple but important case: if the environment of the emitter has sufficiently high symmetry, such as in the case of free atoms, angular momentum conservation requires that the angular momentum of the photon is equal to the difference between the angular momenta of the two atomic states:

$$s=J_{e}-J_{g}.$$

Here, s is the photon angular momentum, while $J_{e,\;g}$ are the angular momenta of the electronically excited and electronic ground states of the atom.

In systems with lower symmetry, the angular momentum may not be a conserved quantity, and the polarization of the photons may not depend on the spin of the participating states. Even in those cases, however, it may be possible to infer the angular momentum state of the quantum system from some properties of the measured fluorescence. A good example is the NV system in diamond, which will be discussed in Sect. [Sec Ch1.S4.SS3]. Here, the number of scattered photons is a good indicator of the angular momentum state: if the system is initially in the $m_{S}=0$ state, the photoluminescence rate is typically 20 % higher than for the $m_{S}=\pm 1$ states [Bibr bib1.bibx27].

Changes in the rate of spontaneous emission cannot only be induced by driving spin transitions with MW or RF fields, but also by tuning the energy levels with a static magnetic field. As an example, a magnetic field can tune the energy of long-lived states (e.g. due to a spin-forbidden transition to the ground state) to match the energy of a state with a short radiative lifetime. As a result, even small symmetry-breaking terms mix the two (near-)degenerate states, resulting in significant increase in the photoemission rate and/or the polarization of the PL. Since the coupling terms mix the two levels, the degeneracy is avoided and the system goes through a level anticrossing (LAC). Measuring these resonances (see e.g. [Bibr bib1.bibx8]) corresponds to a magnetic resonance experiment without an alternating (ac) field driving the transition.

#### 2.4.3 Hanle effect

In the presence of a magnetic field, angular momentum in the system is no longer conserved, but undergoes Larmor precession. This affects the polarization of the emitted photons. Under conditions of continuous optical pumping and in the presence of relaxation, the system evolves towards a stationary state that can be characterized by the polarization of the emitted radiation. This effect is known as the Hanle effect [Bibr bib1.bibx44] and is used extensively for measuring relaxation rates, coupling strengths, and magnetic resonance, mostly in atomic vapours and in semiconductors.

To discuss this effect, we focus on a specific system, where the electronically excited state has no orbital angular momentum ( l=0 ) and spin s=1/2 , and we only discuss the excited state of this spin- 1/2 system, which may correspond to an excited state of an atom or the conduction band of a semiconductor like GaAs. Under steady-state illumination, the stationary state is determined by the interplay between the generation of new spins (along the direction of propagation of the incident laser), relaxation (reducing the polarization and the number of charge carriers), and Larmor precession in the magnetic field. These contributions can be summarized by an equation of motion

2 $$\frac{d\rho }{dt}=-\frac{i}{\hbar }\left[H,\rho \right]-\Gamma_{r}\rho -{\hat{\Gamma }}_{s}+\hat{P^{\prime }},$$

where $\Gamma_{r}$ is the radiative decay rate, and the spin relaxation tensor ${\hat{\Gamma }}_{s}$ , the Hamiltonian H , and the pumping matrix $\hat{P^{\prime }}$ are

$$\begin{array}{cc} {\hat{\Gamma }}_{s} & =\Gamma_{1}\left(\begin{array}{cc} \frac{\rho_{11}-\rho_{22}}{2} & \rho_{12}\\ \rho_{21} & -\frac{\rho_{11}-\rho_{22}}{2} \end{array}\right)\\ H & =\hbar \gamma_{e}B\cdot S\\ \hat{P^{\prime }} & =\left(\begin{array}{cc} P & 0\\ 0 & 0 \end{array}\right). \end{array}$$

Here, the direction of the laser beam was chosen to be along the z axis, so the absorption of a photon creates electron spins along the +z axis. P is the rate at which the laser beam generates electron spin density in the excited state, and we have assumed that the spin relaxation rate $\Gamma_{1}$ is the same for all three components of the spin, $\Gamma_{1}=1/T_{1}=1/T_{2}$ .

To solve the equation of motion, it is often more convenient to choose the z axis along the magnetic field direction. Writing θ for the angle between the directions of the magnetic field and the laser beam, the pumping matrix is then

$$\hat{P}=\frac{P}{2}\left(\begin{array}{cc} 1+\mathrm{cos}\theta & \mathrm{sin}\theta \\ \mathrm{sin}\theta & 1-\mathrm{cos}\theta \end{array}\right).$$

Solving this for the stationary condition, one obtains the steady-state electron density in the excited state as the trace of the density operator,

$$\rho_{11}+\rho_{22}=\frac{P}{\Gamma_{r}},$$

and the x , y , and z components of the polarization vector of the electron spin are

3 $$\begin{array}{lcl} \rho_{12}+\rho_{21} & = & P\frac{\mathrm{sin}\theta \left(\Gamma_{r}+\Gamma_{1}\right)}{{\left(\Gamma_{r}+\Gamma_{1}\right)}^{2}+\Omega_{L}^{2}}\\ -i\left(\rho_{12}-\rho_{21}\right) & = & P\frac{\Omega_{L}}{{\left(\Gamma_{r}+\Gamma_{1}\right)}^{2}+\Omega_{L}^{2}}\\ \rho_{11}-\rho_{22} & = & \frac{P\mathrm{cos}\theta }{\Gamma_{1}+\Gamma_{r}}. \end{array}$$

**Figure 8 Ch1.F8:**
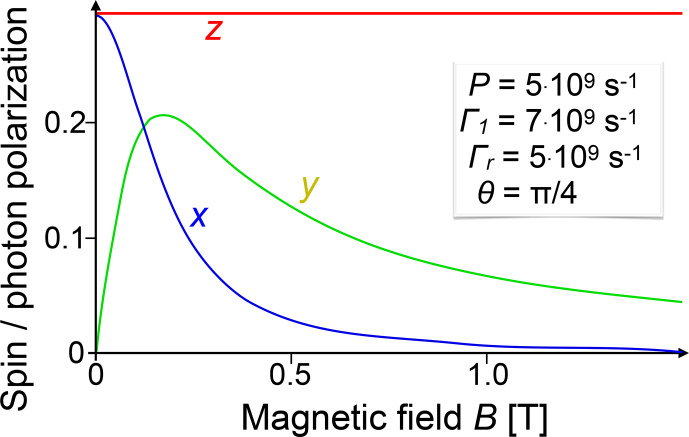
Polarization of the emitted photons as a function of the magnetic field B applied along the z axis. The laser is incident in the x – z plane, at an angle of 45 ${}^{\circ }$ from the magnetic field. The remaining parameters are shown in the figure.

For simplicity, we assume here that the polarization of the photoemission depends only on the spin polarization of the electrons in the excited state. Figure [Fig Ch1.F8] shows the dependence of the spin polarization when the laser beam is incident in the x – z plane and the magnetic field is applied along the z axis. While the z component is not affected by the magnetic field, the transverse components show a typical absorption/dispersion behaviour. The width of the line is given by the electron spin relaxation rate $\Gamma_{1}$ and the radiative lifetime $\Gamma_{r}$ , which can be measured independently [Bibr bib1.bibx86]. This was first discussed by Hanle in the context of atomic fluorescence and observed in semiconductors by Parsons [Bibr bib1.bibx81]. It is still used in the resonance fluorescence from atomic vapours, for the detection of magnetic fields or nuclear spins in zero and ultra-low fields, as discussed in Sect. [Sec Ch1.S3.SS3], and for measuring spin dynamics in semiconductors, as discussed in Sect. [Sec Ch1.S5.SS4].

#### 2.4.4 Coherent Raman scattering

Scattering of light by spin systems cannot only occur spontaneously, but also in a coherent manner. A good example is coherent Raman scattering, where a coherent electromagnetic field (typically a laser field) interacts with the system under study to generate a second field, whose frequency and possibly momentum and polarization are different from the incident field. While the cross sections of such processes may be relatively small, the scattered field depends strongly on the system properties and therefore carries a significant amount of information about the system.

**Figure 9 Ch1.F9:**
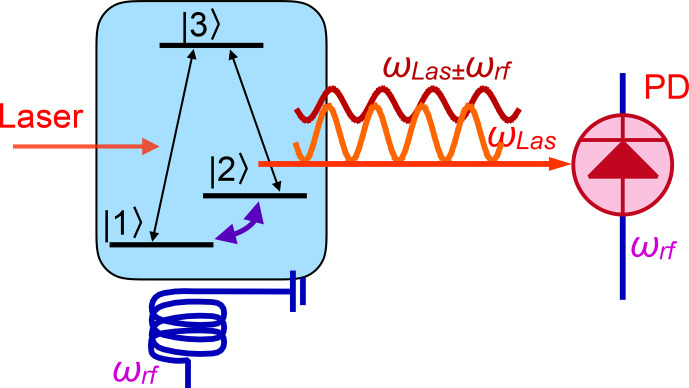
Basic principle of coherent Raman scattering.

Figure [Fig Ch1.F9] illustrates the process for a basic three-level system, where a superposition of the two ground-state levels |1〉 and |2〉 corresponds to a precessing magnetization, which can, in principle, be detected via the voltage induced in an RF coil. In the case of detection through a laser field, the incident laser is (near-)resonant with transition |1〉↔|3〉 . It therefore generates a coherent superposition of these two levels:

$$|1\rangle \overset{\text{Laser}}{⟶}\frac{1}{\sqrt{2}}\left(|1\rangle +|3\rangle \right),$$

which will then evolve as

$$\frac{1}{\sqrt{2}}\left(|1\rangle +|3\rangle e^{-i\omega_{13}t}\right),$$

where $\omega_{13}$ is the energy difference between the states |3〉 and |1〉 , divided by ℏ . This coherent superposition state corresponds to an electric dipole oscillating at the laser frequency $\omega_{L}$ . Due to the coherence between levels |1〉 and |2〉 , however, it simultaneously generates coherence in the transition |2〉↔|3〉 ,

$$\frac{1}{\sqrt{2}}\left(|1\rangle +|2\rangle \right)\overset{\text{Laser}}{⟶}\frac{1}{2}|1\rangle +\frac{1}{\sqrt{2}}|2\rangle +\frac{1}{2}|3\rangle ,$$

which represents a coherent superposition of all three states and therefore contains coherence in all three transitions. It evolves as

$$\frac{1}{2}|1\rangle +\frac{1}{\sqrt{2}}|2\rangle e^{-i\omega_{12}t}+\frac{1}{2}|3\rangle e^{-i\omega_{13}t}.$$

The coherence in the three transitions therefore oscillates with frequencies $\omega_{12}$ , $\omega_{13}$ , and $\omega_{23}=\omega_{13}-\omega_{12}$ . While $\omega_{12}$ is in the RF or MW range, the frequencies $\omega_{13}$ and $\omega_{23}$ are optical frequencies. Accordingly, the system emits two optical fields with these frequencies, with optical polarizations that are determined by the dipole moments of the two transitions. Since this is a coherent process (as opposed to spontaneous emission), the emitted field is phase-locked to the two sources, which is essential for applying heterodyne detection techniques. In the case of the Λ -type system shown in Fig. [Fig Ch1.F9], the spin coherence is part of the electronic ground state and the resulting linewidth can approach the natural linewidth, not being limited by the lifetime of the connected electronically excited state or by the laser jitter.

While this process shares properties of optical and magnetic resonance, it also shows some unique features. As an example, in a continuous wave (CW) experiment, where the transitions |1〉↔|2〉 and |1〉↔|3〉 are irradiated continuously, the amplitude

$$s_{23}\propto \mu_{ij}\mu_{jk}\mu_{ki}$$

of the emitted field in the transition |2〉↔|3〉 is given by the product of all three transition amplitudes [Bibr bib1.bibx106]. One consequence of this tri-linearity is that, on many occasions, there is interference between different signal contributions, which can lead to cancellations [Bibr bib1.bibx54]. In particular, many systems show a symmetry between the Stokes scattering pathway, where the frequency of the scattered field is equal to the difference $\omega_{L}-\omega_{\mathrm{RF}}$ between the frequency $\omega_{L}$ of the laser field and the RF $\omega_{\mathrm{RF}}$ and the anti-Stokes field, whose frequency is the sum $\omega_{L}+\omega_{\mathrm{RF}}$ . If these signals have opposite amplitudes, they cancel and the expected resonance line appears to be missing. This can be avoided by suppressing the symmetry, e.g. by adding a pump laser beam that reduces the population difference across one of the two transitions or increases that of the second transition [Bibr bib1.bibx72].

### 2.5 Sensitivity limits

As discussed above, optical methods can increase the population difference of spin systems by many orders of magnitude, and they increase the detection sensitivity compared to inductive detection. This is not only due to the higher signal energy of optical photons, but also to the virtual absence of thermal noise at optical frequencies. A third reason for the increased sensitivity is that laser irradiation can polarize the spins much faster: depending primarily on the laser intensity, complete polarization of the spin system may require less than 1 µs [Bibr bib1.bibx95]. Since optical detection directly measures the magnetization, in contrast to pick-up coils that measure its time derivative, the detection sensitivity is independent of the resonance frequency. It is therefore possible to perform experiments at low or vanishing fields with the same detection efficiency as at high fields.

#### 2.5.1 Transmission

In a transmission experiment described in Sect. [Sec Ch1.S2.SS4.SSS1], the relevant noise has mostly three contributions: (i) laser intensity fluctuations, (ii) shot noise of the laser beam, and (iii) thermal noise of the detection. The first type of noise can be reduced significantly, e.g. by the balanced detection scheme shown in Fig. [Fig Ch1.F6]. The thermal noise of the detector can be reduced by technical measures, such as cooling. However, it is not always possible to reduce this contribution to insignificant levels. The shot noise of the laser, finally, is not eliminated by the balanced detection scheme, since it is anti-correlated between the two channels. Keeping this contribution low, relative to the signal, requires one to use high laser power, which is naturally limited by the properties of the sample: not only overheating can be a problem, but also the dynamics of the system that one tries to detect may be modified at high laser intensity.

#### 2.5.2 Fluorescence

The sensitivity of fluorescence detection varies significantly between systems. Ideally, it would be possible to determine the spin state of a particle by measuring the polarization of a single fluorescence photon. Such a measurement could be repeated immediately after detecting the photon, which allows a readout with very high certainty in a time below 1 µs . In real systems, however, the actual readout time is significantly longer. In the case of a diamond NV centre, for example, the rate at which photons are detected is typically of the order of $10^{5}$ s ${}^{-1}$ . Furthermore, the information is carried here only by the rate at which photons are emitted (not by the polarization), and this rate differs between the spin states by some 20 % [Bibr bib1.bibx27]. Accordingly, it typically takes up to 1 ms to determine the state of a single spin with sufficiently high certainty.

#### 2.5.3 Single spin detection

Detecting the signal of single spins [Bibr bib1.bibx108] mostly requires the suppression of unwanted signal contributions from other sources, such as scattered laser light or fluorescence from other sources. Consider e.g. the common situation of a NV centre in diamond, where a 0.1 mW laser beam is used for excitation, which corresponds to some $2.7\times 10^{15}$ photons per second. From a single centre, we typically obtain some $10^{5}$ fluorescence photons per second. Accordingly, the fraction of laser photons that can be allowed to reach the detector must be less than $\approx 10^{-12}$ to avoid significant degrading of the signal. This is typically achieved by a combination of spectral filtering, where the higher-energy laser photons are reflected or absorbed, while the lower-energy fluorescence photons are transmitted, and spatial filtering, e.g. by the pinhole of a confocal microscope.

Figure [Fig Ch1.F10] shows the photon count rate from a single NV centre measured as a function of position. The width of the peak (HWHH), which is determined by the resolution of the instrument, is 113 nm. This diffraction-limited focus is still large compared to the size of the centre ( ≈0.1 nm), but it suppresses background signals from the rest of the sample and allows one therefore to detect a single centre by accumulating photons for ≈1 ms. Other examples of experiments with single spins are discussed in the subsequent sections.

**Figure 10 Ch1.F10:**
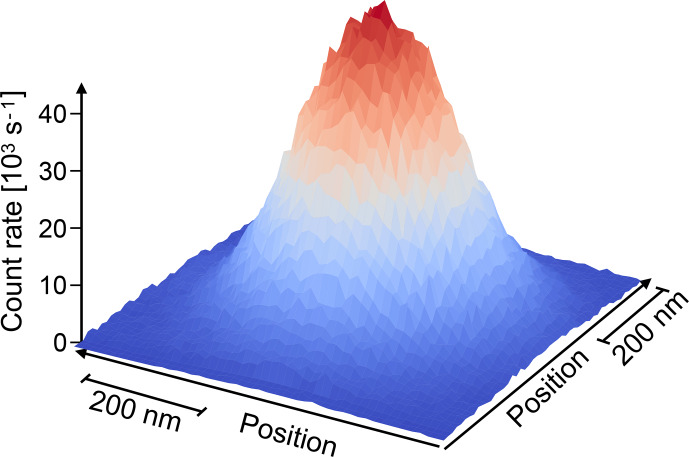
Photon count rate from a single NV centre in diamond measured as a function of position.

## 3 Atomic and molecular systems

Atomic vapours are very useful systems for studying the basics of optically detected magnetic resonance. Very often it is possible to neglect spatial degrees of freedom and interactions between atoms. In these cases, the Hilbert space of the system can be limited to the angular momentum degrees of freedom plus a small number of electronic states that interact with the laser beam. We therefore start the discussion with these systems and move to more complicated systems with higher practical relevance in the following chapters.

### 3.1 Detection of electron spin

Perhaps the most direct possibility for detecting magnetic resonance optically consists in using a high-resolution optical spectrometer and monitoring the intensity of two resonance lines from a Zeeman doublet.

Figure [Fig Ch1.F11] shows the basic principle: the system consists of an electronic ground state and an electronically excited state, both of which can have two possible angular momentum states. As discussed in Sect. [Sec Ch1.S2], optical transitions can only occur between pairs of states that allow conservation of angular momentum; in the example shown here, circularly polarized light interacts either with the blue or the red transition. A magnetic field shifts the ground as well as the electronically excited states in different directions and therefore lifts the degeneracy of the resonance lines. In the example shown in the figure, the red transition is shifted to lower frequencies, while the blue transition shifts to higher frequencies. If a magnetic resonance experiment changes the relative occupation numbers of these angular momentum states, it affects the amplitudes of the resonance lines in different ways. A resonant excitation of the spin transitions can therefore be monitored through the change in the amplitudes of the resonance lines or, if they are not completely resolved, through an asymmetry in the spectrum or a shift of the mean frequency [Bibr bib1.bibx34].

**Figure 11 Ch1.F11:**
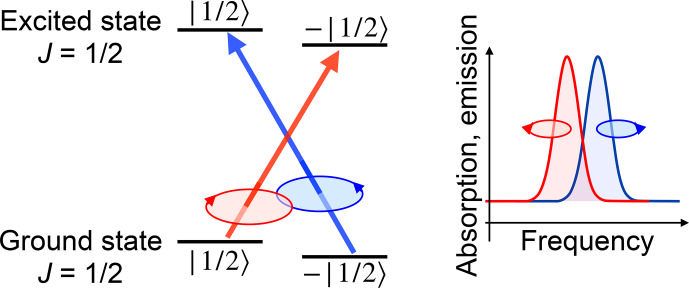
Energy level system and resonance lines of a Zeeman doublet.

In many systems, even the natural linewidth of the relevant transitions is large compared to the difference in their resonance frequencies, so the overlap is too large to allow for their separation in a spectrometer. Nevertheless, it is possible to monitor differences in the populations of the spin substates through their effect on absorption, dispersion, and emission of optical transitions between these states. As discussed in Sect. [Sec Ch1.S2.SS4.SSS1], the polarization of light transmitted through a resonant medium depends on the spin polarization in the medium. It can thus be used to monitor the spin polarization.

Atomic vapours, particularly of alkali metals, are the simplest systems for discussing and testing these effects. It is therefore not surprising that they were also the first systems where the effect was studied [Bibr bib1.bibx51]. In this case, the complex index of refraction (i.e. absorption as well as dispersion) [Bibr bib1.bibx84] depends linearly on the spin polarization of the ground state. It is therefore possible to measure a component of the spin polarization by transmitting a laser beam in that direction through the sample and detecting either the circular dichroism or the Faraday rotation of the light – typically with differential detection schemes like those discussed in Sect. [Sec Ch1.S2.SS4.SSS1].

Such experiments cannot only probe the bulk of an atomic vapour; they can also be used to selectively study interfaces, e.g. by reflecting a laser beam from an interface between glass and vapour [Bibr bib1.bibx97] or from the surface of a crystal [Bibr bib1.bibx66], in order to selectively study only a few atomic layers close to the surface.

All these experiments are typically performed at low magnetic fields, where the Zeeman interaction for the electronic as well as nuclear angular momentum is small compared to the hyperfine interaction between them. Accordingly, the relevant optical properties of the system cannot be related to either of these spins individually, but to the total angular momentum

F=J+I=L+S+I,

where I represents the nuclear spin, S the electron spin, L the orbital angular momentum of the electron, and F the total angular momentum of electron and nucleus. Only when the electronic Zeeman interaction is stronger than the hyperfine interaction can the two angular momenta be considered independently.

### 3.2 Detection of nuclear spins

Since the nuclear spin does not couple to the electric dipole moment of optical transitions, direct optical detection of NMR is not as straightforward as for electron spins. However, since nuclear spins interact with electron spins, indirect detection schemes are possible and have been explored. Here, we can only discuss a few of the possible schemes, and we focus on NMR transitions in electronic ground states, which is probably the most relevant situation.

We first consider the case where the electronic ground state has unpaired electrons, i.e. S>0 . In this case, the hyperfine interaction is typically the dominant interaction for the nuclear spin. It is then strongly coupled to the electron angular momentum, as discussed at the end of Sect. [Sec Ch1.S3.SS1], and can be detected exactly in the same way as the electron spin.

**Figure 12 Ch1.F12:**
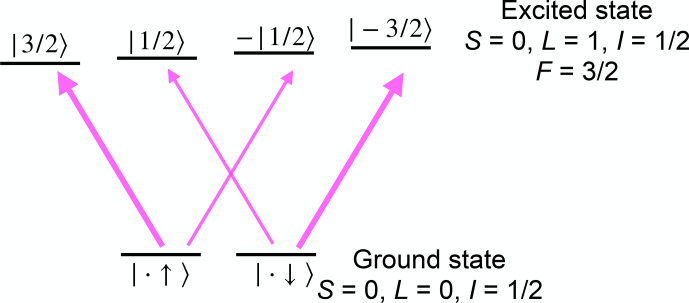
Detection of a nuclear spin state in a system with vanishing electron spin in the ground state. The transition strength of the thinner arrows is $\sqrt{3}/3\approx 0.58$ times that of the thicker ones.

The situation is less straightforward in systems where the electronic angular momentum vanishes, J=0 . In this case, F=I and the interaction with magnetic fields is the same as in other diamagnetic systems. However, the transition matrix elements of optical transitions still depend on the state of the nuclear spin. Figure [Fig Ch1.F12] shows the basic principle for a transition between the electronic ground state with L=S=0 and an electronically excited state with L=1 , S=0 . In this case, angular momentum selection rules imply that only the transitions indicated by arrows in Fig. [Fig Ch1.F12] have non-vanishing transition moments for light propagating along the quantization axis. The transition strength of the thinner arrows is $\sqrt{3}/3\approx 0.58$ times that of the thicker ones. In addition, the excited states interact with a magnetic field much more strongly than the nuclear spin, and therefore they split in a magnetic field, so these allowed transitions are non-degenerate. Accordingly, the nuclear spin state has a significant influence on the transition probabilities, which allows one to determine the spin state from either absorption or emission measurements [Bibr bib1.bibx100]. Enhancing the interaction between the radiation field and the atomic system, e.g. by a resonant cavity, even allows one to detect single nuclear spins in such a system [Bibr bib1.bibx100].

### 3.3 Detection of NMR by optical magnetometry

Light transmitted through atomic vapours provides a very sensitive detector for magnetic fields, as discussed in Sects. [Sec Ch1.S2.SS4.SSS1] and [Sec Ch1.S3.SS1]. Accordingly, this type of measurement can be used to detect magnetization from nuclear spins – either inside the optically active medium or outside. The first case (nuclear spins in the active medium) is the typical situation for magnetic resonance of noble gases, in particular ${}^{3}\mathrm{He}$ and ${}^{129}\mathrm{Xe}$ .

Figure [Fig Ch1.F13] shows the basic setup for optically detected NMR of noble gas atoms in an optical magnetometer. The pump laser beam generates spin polarization of alkali atoms, typically rubidium (Rb). During collisions with the noble gas atoms (typically ${}^{129}\mathrm{Xe}$ or ${}^{3}\mathrm{He}$ ), part of their spin polarization is exchanged with the nuclear spins of the noble gas atoms, which results in significant nuclear spin polarization after a large number of collisions. The nuclear as well as the electronic spins evolve in the total magnetic field, which includes contributions from an external bias field (which may be zero), the magnetization of the Rb atoms, and the nuclear spin magnetization of the noble gas atoms. The resulting polarization of the Rb atoms is detected through their effect on the polarization of the transmitted probe beam. A change in any component of the total magnetic field is therefore observed as a change in the signal from the polarization selective detector. This allows optical detection also of the nuclear spins of the noble gas atoms [Bibr bib1.bibx85].

**Figure 13 Ch1.F13:**
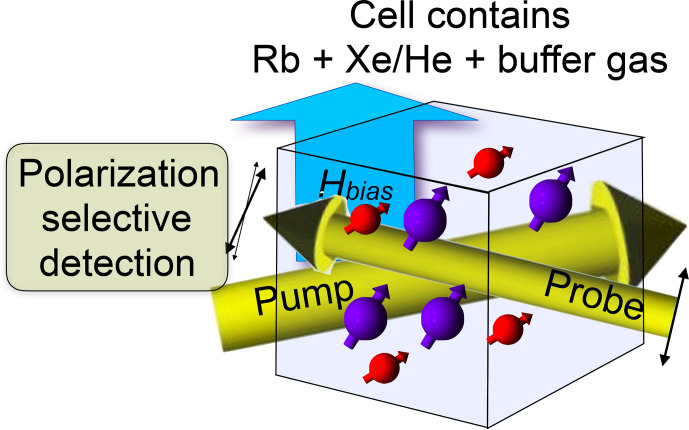
Setup for zero- and low-field NMR in an optical magnetometer based on alkali atomic vapour.

Since the nuclear spins in this system do not interact directly with the laser beam, they can be spatially separated from the Rb magnetometer to allow detection of spin species that are not compatible with an atomic vapour [Bibr bib1.bibx19]. The detection sensitivity of atomic-vapour-based magnetometers is given by the magnetic flux, rather than its derivative with respect to time (as in inductive detectors). Accordingly, it does not increase with the Larmor frequency and is often higher at low frequencies and thus low fields. This makes it a particularly attractive tool for measuring NMR spectra in “ultra-low fields”, where the Zeeman effect is a small perturbation of the zero-field Hamiltonian, which is usually dominated by J couplings for liquid-state NMR [Bibr bib1.bibx4].

The Hanle effect discussed in more detail in Sect. [Sec Ch1.S2.SS4.SSS3] uses a closely related effect: in these systems, the spins of the electrons in the excited states undergo Larmor precession in a magnetic field that is given by the sum of the externally applied magnetic field and an effective field due to the average interaction with a large number of nuclear spins, which is known as the nuclear field.

## 4 Dielectric solids

### 4.1 Rare-earth ions

Dielectric crystals hosting rare-earth ions (REIs) have been studied by optical spectroscopy for many years. The main motivation for these studies derives from the relatively narrow transitions between different electronic states which differ mostly with respect to the configurations of their f electrons. The transitions between these states are therefore “forbidden”; i.e. they have small transition dipole moments and long lifetimes. Furthermore, the electrons are relatively well shielded from perturbations by charged defects, resulting also in a relatively small inhomogeneous broadening of the transitions.

**Figure 14 Ch1.F14:**
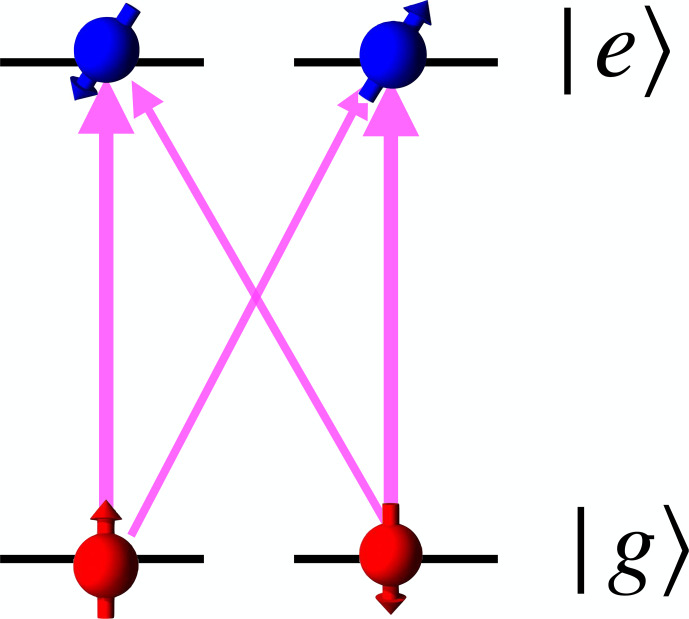
Eigenstates of the nuclear spin in the ground and electronically excited states of a rare-earth ion. The quantization axes (indicated by the direction of the arrows) of ground and excited states have different orientations not along the magnetic field. The pink arrows between the ground and excited states indicate optical transitions, with the widths representing different transition dipole moments.

The nuclei of many rare-earth isotopes have non-zero spin, which interact with external magnetic fields as well as with the angular momenta of the electronic system. One of the consequences is that the nuclear spin eigenstates of different electronic states are not identical. Figure [Fig Ch1.F14] shows the situation for a nuclear spin I=1/2 . Due to the anisotropic hyperfine interaction, the quantization axes of the nuclear spin in the ground and electronically excited states have different orientations, and they depend on the strength of the magnetic field. In the figure, the quantization axes are marked schematically by the orientation of the spins. As the system undergoes electronic excitation, the new state has overlap with both nuclear spin states, and accordingly the transition dipole moments for all four possible transitions are non-zero.

This is a very important precondition for the optical excitation and detection of NMR transitions by coherent Raman scattering (CRS). As discussed in Sect. [Sec Ch1.S2.SS4.SSS4], Raman excitation requires that two optical transitions sharing one energy level have non-vanishing transition dipole moments. According to Fig. [Fig Ch1.F14], this is fulfilled for V-type as well as Λ -type transitions in REI systems, which allows one to use CRS for studying ground as well as excited states.

Figure [Fig Ch1.F15] shows two examples of NMR spectra of ${}^{141}\mathrm{Pr}$ , a typical rare-earth isotope with a nuclear spin of 5/2 . For these experiments, it is added as a dopant to the host crystal YAlO ${}_{3}$ [Bibr bib1.bibx55]. The upper spectrum shows the NMR transitions when the ion is in the electronically excited ${}^{1}D_{2}$ state, while the lower spectrum shows the same transitions for the ${}^{3}H_{4}$ electronic ground state. The large differences in the transition frequencies originate from the different hyperfine interactions, which depend on the electronic configuration. Since the observed signal is proportional to the product of the transition dipole moments of all three transitions, it can be positive or negative. Under the conditions used for the spectra of Fig. [Fig Ch1.F15], the resonance lines of one crystallographic site are positive and those for the second site are negative.

**Figure 15 Ch1.F15:**
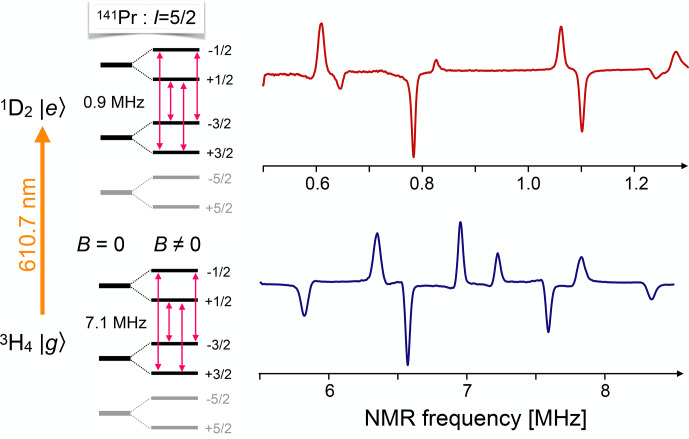
Energy levels and NMR spectra of ${}^{141}\mathrm{Pr}$ in the ground and electronically excited states of ${\mathrm{Pr}}^{3+}$ in a YAlO ${}_{3}$ host crystal, measured by coherent Raman scattering. The grey parts of the level scheme do not contribute to the spectra shown in the figure, but are included for completeness. The four positive lines correspond to the transitions of one crystallographic site, as marked in the level scheme. The four negative lines belong to the corresponding transitions in a different crystallographic site.

One of the most promising applications of optically excited and detected magnetic resonance in rare-earth ions is the possibility of using these materials as memories for quantum states [Bibr bib1.bibx102]. This is an important prerequisite for many emerging quantum technologies, such as quantum communication. Quantum states can be stored in the relatively long-lived electronic states of the rare-earth ions, but transferring them into nuclear spin degrees of freedom can extend the lifetime by several orders of magnitude to the range of seconds [Bibr bib1.bibx65] and, for custom-designed crystals, even to several hours [Bibr bib1.bibx113] – by far the longest lifetime of a quantum memory measured so far.

The experimental techniques for studying these materials include transmission as well as fluorescence experiments, CW, as well as time-resolved experiments. Pioneering work on these systems was performed, e.g. in the group of Brewer, which demonstrated Raman-heterodyne detection, pulsed as well as CW on ${}^{141}\mathrm{Pr}$ ions substitutionally doped into a LaF ${}_{3}$ crystal [Bibr bib1.bibx70]. Excitation of the spin transitions can be performed by RF fields as well as purely optically, e.g. with a bichromatic laser field that excites the spin transition via a coherent Raman process [Bibr bib1.bibx15]. The two frequency components of the laser field should then be separated by the magnetic resonance transition frequency.

### 4.2 Transition metal ions

Transition metal ions have optical resonance lines that are much broader then rare earth ions and depend much more on the environment of the ions. This makes their use in optical detection more challenging but also potentially more rewarding. Transition metal ions are essential components of many biologically relevant molecules.

The most popular test system in this category is certainly ruby, i.e. ${\mathrm{Cr}}^{3+}:{\mathrm{AlO}}_{3}$ [Bibr bib1.bibx34]. Figure [Fig Ch1.F16] shows the energy level system of the electronic ground state and one of the excited states. The transition between the ground state and the ${}^{2}E$ excited state is known as the $R_{1}$ transition (or $R_{2}$ transition for a nearby state). Using ruby as a test bed, [Bibr bib1.bibx34] explored several methods for measuring magnetic resonance in the electronic ground – as well as in excited states. Their approach compared the measurement of circular polarization, selective reabsorption, and high-resolution spectrometry. The same system was also studied by purely optical methods like photon-echo modulation [Bibr bib1.bibx99].

**Figure 16 Ch1.F16:**
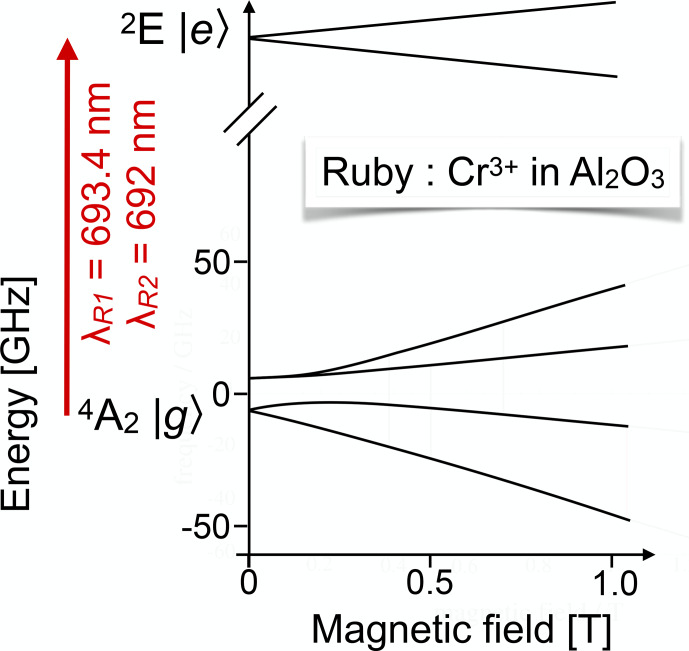
Partial energy level scheme of ${\mathrm{Cr}}^{3+}$ in ruby for the states involved in the $R_{1}$ and $R_{2}$ transitions.

More challenging but also more interesting in terms of the potential information are measurements on biological macromolecules like metalloproteins. In these systems, the optical detection approach provides more information than classical inductive detection, since it relates the magnetic anisotropy to the electronic and molecular structure of the molecule [Bibr bib1.bibx17]. While the analysis of these correlations is not trivial, it allows one to obtain detailed information on the electronic and atomic structures of the molecules in frozen solutions, sometimes circumventing the need for growing single crystals.

### 4.3 The NV centre of diamond

One of the most popular systems for testing magnetic resonance with single spins is the NV centre in diamond [Bibr bib1.bibx67]. Figure [Fig Ch1.F17] shows on the left the structure of the centre: one carbon of the diamond lattice is replaced by a nitrogen atom, while one of its nearest neighbours is missing: the corresponding atom is replaced by a vacancy. The centre is most useful in the negatively charged state (NV ${}^{-}$ ), which contains a total of six electrons: three from the dangling bonds next to the vacancy, two from the lone pair at the nitrogen, and the single additional electron that has been captured from one of the available donors. The ${}^{3}A_{2}$ ground state of these six electrons has spin S=1 , and it can be optically excited into a ${}^{3}$ E state. This transition does not normally change the spin state and, in most cases, the centre falls back into the same ground state. However, there is a finite probability for the centre to undergo ISC into the singlet manifold. This probability is significantly larger for centres in the $m_{S}=\pm 1$ state than in the $m_{S}=0$ state. From the singlet manifold, the system falls back into the ground state, but during this process, the spin state becomes randomized. The overall effect is that the $m_{S}=0$ state becomes significantly more populated than the other states. This process does not require polarized light and can be easily observed in conventional ESR experiments, where irradiation with white light results in a strongly enhanced ESR spectrum [Bibr bib1.bibx64].

**Figure 17 Ch1.F17:**
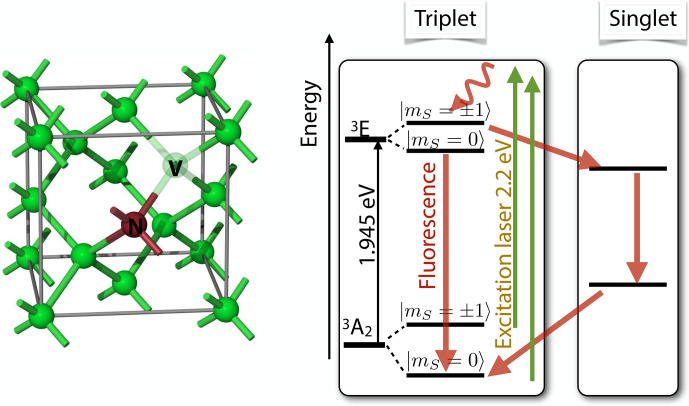
Structure of the nitrogen-vacancy (NV) defect centre in diamond and energy level system. Optical excitation results in spin-dependent ISC to the singlet state, which occurs predominantly for the $m_{S}=\pm 1$ states and therefore results in preferential population of the $m_{S}=$ state of the ${}^{3}A_{2}$ ground state.

When the system takes the “detour” through the singlet state, this requires significantly more time than the direct path back to the ground state, and it does not emit a photon during this cycle. Accordingly, the centres generate some 20 % fewer photons when they are initially in the $m_{S}=\pm 1$ state. Counting the number of photons emitted per unit time is therefore a simple way of detecting the spin state of the system at the start of the counting period. This readout is destructive, as the system is forced into the $m_{S}=0$ state by the readout process, but it can be used to detect e.g. magnetic resonance spectra [Bibr bib1.bibx27].

Figure [Fig Ch1.F18] shows two examples of ESR spectra. The upper one shows three transitions, two of them corresponding to “allowed” $\Delta m_{S}=\pm 1$ transitions and the central one to the “forbidden” $m_{S}=-1↔m_{S}=+1$ transition [Bibr bib1.bibx74]. All three transitions are split by the hyperfine interaction with the ${}^{14}$ N ( I=1 ) nuclear spin. For the $\Delta m_{S}=\pm 2$ transition, the splitting is twice as large as for the $\Delta m_{S}=\pm 1$ transitions.

**Figure 18 Ch1.F18:**
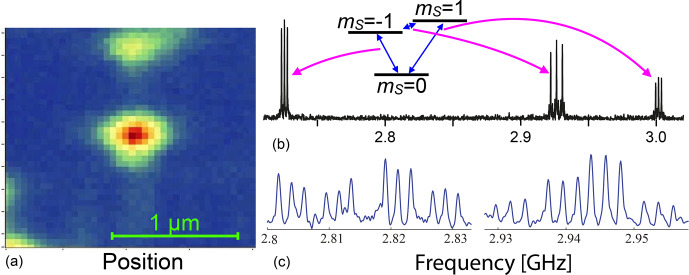
**(a)** Scanning confocal image of a single NV centre. **(b, c)** Optically detected ESR spectra of two different NV centres showing hyperfine interaction with the ${}^{14}N$ **(b)** and the ${}^{14}N$ plus a ${}^{13}C$ **(c)** nuclear spin.

The lower trace shows the ESR spectrum from a different centre, where one of the neighbouring atoms is a ${}^{13}C$ nuclear spin, which also has a hyperfine interaction with the electron. While the interaction with a I=1/2 nuclear spin normally leads to a splitting into a doublet (of the triplet due to the ${}^{14}N$ interaction), it results here in a quartet [Bibr bib1.bibx83]. This is a consequence of the different orientations of the nuclear spin quantization axes in the different electron spin states. As shown in Fig. [Fig Ch1.F14], this implies that all four possible transitions become (partly) allowed.

The hyperfine interaction allows one to observe not only EPR transitions, but also nuclear spin transitions – in many cases even without applying RF pulses [Bibr bib1.bibx112]. While the spectra shown here are all associated with magnetic resonance of electronic and nuclear spins that are part of the NV centre, it is also possible to use NV centres as sensors for indirectly detecting more remote spins, either electronic or nuclear ones [Bibr bib1.bibx5].

### 4.4 Silicon carbide

Silicon carbide is a material that is closely related to diamond: its structure can be derived from diamond by replacing alternating carbon sites with silicon. It also shares other properties like a large band gap and high mechanical strength. There are also major differences; in particular, there is not just a single structure, but the material has a large number of polytypes, where successive SiC layers follow different stacking patterns. In each polytype, several active spin centres have been described (see e.g. [Bibr bib1.bibx33]), mostly silicon and carbon vacancies, as well as divacancies. Depending on their charge state, they have a spin of 1/2 , 1 , or 3/2 (in units of ℏ ).

Figure [Fig Ch1.F19] shows on the left-hand side the energy level scheme of a typical spin centre in SiC: a ${\mathrm{Si}}^{-}$ vacancy in the 6H polytype. Depending on the site, there are several such centres – the one shown here is the $V_{2}$ centre, which has a zero-field splitting of 128 MHz between the $m_{S}=\pm 1/2$ and $m_{S}=\pm 3/2$ states [Bibr bib1.bibx11]. The right-hand part of the figure shows the ODMR spectrum, measured by the change in photoluminescence when an RF field is applied, as a function of the magnetic field and the RF. The change in the PL intensity is colour-coded according to the scale bar on the right. The experimental data contain signals from the $V_{2}$ centres, whose energy level scheme is shown on the left, as well as from the $V_{1}/V_{3}$ centres, whose zero-field splitting is -28 MHz. The ODMR signal from the $V_{1}/V_{3}$ centres is positive (i.e. the PL increases when an RF field is applied), while the signal from the $V_{2}$ centres is negative [Bibr bib1.bibx90].

**Figure 19 Ch1.F19:**
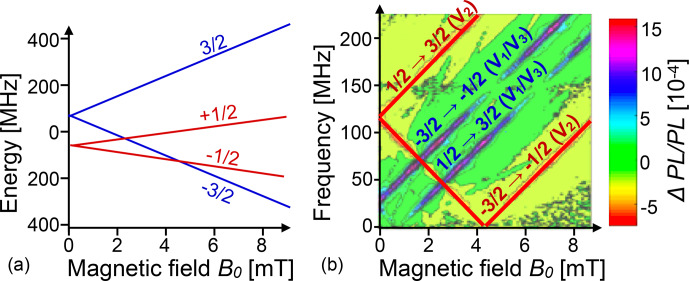
**(a)** Energy level system of the V ${}_{2}$ ${\mathrm{Si}}^{-}$ vacancy in SiC (6H polytype). **(b)** Experimental ODMR spectra as a function of the magnetic field (horizontal axis) and RF (vertical axis). The experimental spectrum contains transitions from V ${}_{1}$ and V ${}_{3}$ vacancies. The colour indicates the relative change in PL.

## 5 Semiconductors

### 5.1 Optical pumping

Optical pumping, i.e. the transfer of angular momentum from photons to electronic and nuclear spins, can follow two distinct paths in semiconductors, depending on whether the photons are absorbed by localized defect states such as deep donors or whether they target directly the delocalized electrons, raising them from the valence to conduction bands. A good example of the work with localized defects is [Bibr bib1.bibx57], which used the 2.2 eV transition of a residual donor in nominally undoped GaN to measure the nuclear spin transitions of both Ga isotopes via optically detected ENDOR.

**Figure 20 Ch1.F20:**
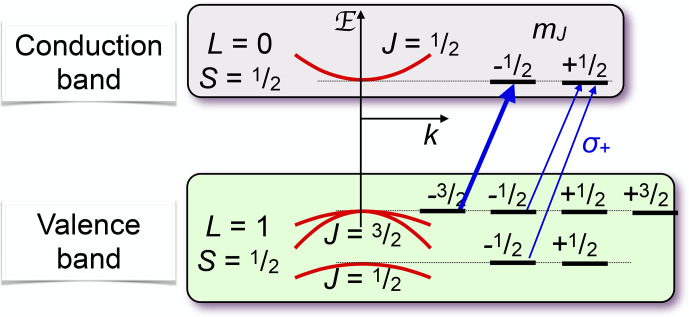
Optical pumping in a direct-band-gap semiconductor. The arrows mark the allowed optical transitions for circularly polarized light.

Figure [Fig Ch1.F20] shows the basic principle of optical pumping via delocalized electrons for a semiconductor with a direct band gap, such as GaAs, where the bottom of the conduction band is at the same linear momentum as the top of the valence band. These materials are particularly useful for light-emitting devices, such as lasers and LEDs. The electronic states in the valence band, which has a p -type character, have total angular momentum J=3/2 , while the states in the conduction band ( s character) have angular momentum $J{}^{\prime }=1/2$ . A circularly polarized laser field therefore couples only the transitions indicated in the figure (or the corresponding mirror image for opposite circular polarization). Optical excitation is most efficient if the photon energy is close to the exciton energy, thereby generating charge carriers in both bands with minimal kinetic energy.

If pumping is done in a magnetic field, the eigenstates of the electron system are the Landau levels, and optical pumping above the band gap must be described in terms of Landau levels [Bibr bib1.bibx71].

The mechanism shown in Fig. [Fig Ch1.F20] requires photon energies at or above the band gap. While this works for all systems, it is also possible to use below-band-gap light for pumping. In this case, the photons are absorbed by localized centres, such as donors, whose energy levels are inside the band gap [Bibr bib1.bibx82]. For some samples, this process is actually more useful, particularly in bulk material, since the absorption coefficient is smaller. The light can then penetrate farther into the sample and achieve more homogeneous pumping.

**Figure 21 Ch1.F21:**
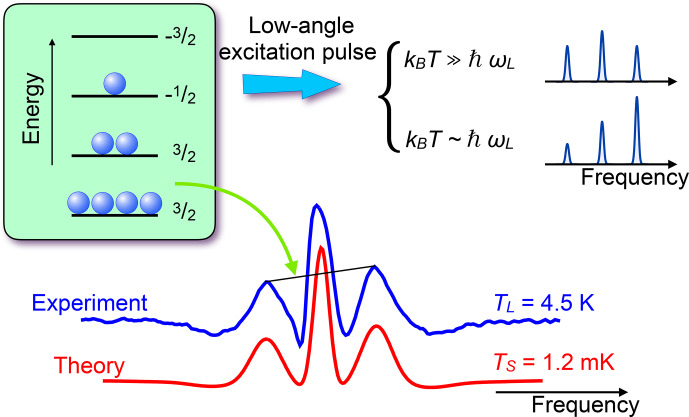
Experimental and simulated NMR spectra of ${}^{75}\mathrm{As}$ in GaAs, measured with a small flip angle. The different amplitudes of the quadrupole satellites are then proportional to the population differences across the corresponding transitions and therefore allow a direct measurement of the spin temperature. The calculated populations of the four levels are 0.123, 0.185, 0.277, and 0.415.

### 5.2 Dynamic nuclear polarization

The hyperfine interaction can exchange polarization between electronic and nuclear spin, resulting in significant polarization of the nuclear spins. Depending on the system, this process can involve different steps. Here, we first discuss a process where the optical irradiation generates electron spin polarization on a localized centre, such as a shallow donor in GaAs [Bibr bib1.bibx79] or an NV centre in diamond [Bibr bib1.bibx27]. A typical experiment on diamond starts with a laser pulse that initializes the electron spin into the $m_{S}=0$ ground state. The electron spin is coupled to the ${}^{14}N$ nuclear spin of the NV centre as well as to many ${}^{13}C$ nuclear spins at random locations in the lattice. The hyperfine interaction with the ${}^{13}C$ nuclear spins reaches a maximum value of ≈130 MHz [Bibr bib1.bibx83] for nuclei located next to the vacancy. For more remote nuclei, it decreases with the third power of the distance. Depending on the interaction strength, the directly coupled nuclear spins also acquire some polarization from the electron spin, but this process can be made much more efficient by the application of microwave fields [Bibr bib1.bibx2]. From the directly coupled nuclear spins, the polarization spreads to the bulk of the crystal through nuclear spin diffusion.

If the charge carriers are delocalized, their polarization can also be transferred to nuclear spins through the Overhauser effect [Bibr bib1.bibx60] or by dynamic nuclear polarization driven by microwave irradiation. The rate at which the nuclear spin polarization builds up is close to the spin-lattice relaxation rate. These rates can vary significantly over different materials and doping levels, but are typically of the order of minutes at low temperature [Bibr bib1.bibx87]. The efficiency of this process depends strongly on the system, but in some cases, it can cool the nuclear spins to very low temperatures, as in the example shown in Fig. [Fig Ch1.F21]. Here the spectrum of ${}^{75}\mathrm{As}$ , which has a spin I=3/2 in GaAs, is split by the quadrupole interaction, which allows one to observe all three transitions separately. If the system is excited by an RF pulse with a small ( ≪π/2 ) flip angle, the amplitudes of the lines are proportional to the population differences, which allows one to measure the spin temperature of the system. For this measurement, the magnetic field was $B_{0}=1.38$ T and the resonance frequency 10.1 MHz. The measured spin temperature is $T_{S}\approx 1.2$ mK, while the sample (lattice) is at a temperature of $T_{L}\approx 4.5$ K.

### 5.3 Nuclear field

In the most important semiconductors, the conduction band is formed by s -type orbitals. The electronic spins in the conduction band therefore interact with the nuclear spins in the material through hyperfine interactions dominated by the Fermi contact term. If the electrons are localized (e.g. near interface fluctuations or impurities such as shallow donors), their wavefunction has a radius of the order of tens of nanometres, which means that at least $10^{5}$ nuclear spins have direct contact with the electron spin. The interaction strength with a single nuclear spin is of the order of some tens of kHz. From the point of view of the electron, this is very small compared to the other interactions. As long as the nuclear spins have thermal polarization, i.e. roughly equal populations of ↑ and ↓ , their effect on the dynamics of the electron spin is therefore small. However, since the number of nuclear spins that interact with the electron is very large, these small contributions can add up to very large effects. The first is a dephasing effect: due to statistical fluctuations (“spin noise”, [Bibr bib1.bibx91]), the electron interacts with a fluctuating environment that can result in dephasing of the electron spin.

If the nuclear spins are polarized (see Sect. [Sec Ch1.S5.SS2]), their interactions do not cancel, but add up, and their combined effect can be very significant and become the dominant interaction for the electrons. For large polarization, the effect is comparable to a magnetic field of the order of several Teslas [Bibr bib1.bibx80]. The effective nuclear field exists for localized as well as mobile electrons, but the effect differs. For metals, the effect was calculated by Overhauser [Bibr bib1.bibx77]. Figure [Fig Ch1.F22] shows the build-up of the nuclear field in GaAs during optical pumping. This effective magnetic field adds to the external field and the evolution of the electron spins is determined by their sum. If one measures the polarization of the photoluminescence as a function of a transverse magnetic field, one therefore observes a shifted Hanle curve whose maximum marks the external field that has the same magnitude but opposite sign as the internal nuclear field.

**Figure 22 Ch1.F22:**
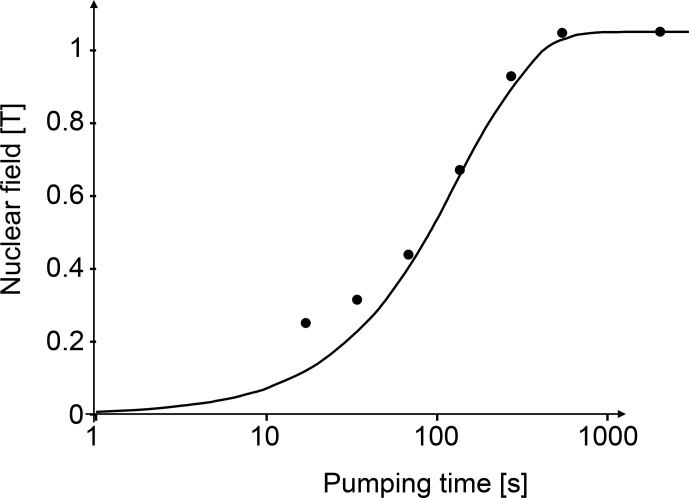
Build-up of the nuclear field during optical pumping.

### 5.4 Detection of NMR via the Hanle effect

The Hanle effect discussed in Sect. [Sec Ch1.S2.SS4.SSS3] is often used to monitor the effect of optical pumping in semiconductors or to detect magnetic resonance transitions. It works well e.g. in direct band-gap semiconductors like GaAs, where the conduction band consists of s -type orbitals and the excited charge carriers do not relax too quickly. The valence band of these materials consists of p -type orbitals and the spin–orbit interaction therefore leads to a relatively fast relaxation of the holes. It is then often a good approximation to consider only the spin state of the electrons in the conduction band.

Figure [Fig Ch1.F23] shows the emission of a photon during recombination. The energy level scheme shown here is that of a quantum well (a very thin layer of GaAs sandwiched between barriers of AlAs), and in the valence band, only the J=3/2 levels are shown. Compared to the level scheme of Fig. [Fig Ch1.F20], which represents the levels of a bulk material, the J=3/2 levels are not degenerate in the quantum well. The situation shown in the figure corresponds to a single electron in the conduction band, in the $J_{z}=-1/2$ state. In the valence band, a single hole exists in the $J_{z}=-3/2$ state, e.g. because it was created there by the absorption of a photon. If the electron and hole recombine to emit a photon, their angular momentum must be carried away by the photon, which is circularly polarized. Accordingly, the spin polarization of the emitted photon directly reflects the polarization of the electron spin, which can be modified by relaxation and by magnetic fields, as discussed in Sect. [Sec Ch1.S2.SS4.SSS3]. When polarized nuclear spins are present, the nuclear field adds to the external field and therefore shifts the Hanle curve: the maximum of the PL polarization is no longer at the zero external field, but at an external field that is exactly equal in magnitude but opposite in direction to the nuclear field. The sum of the external and nuclear fields is thus zero and the electron spin polarization is not degraded [Bibr bib1.bibx28].

**Figure 23 Ch1.F23:**
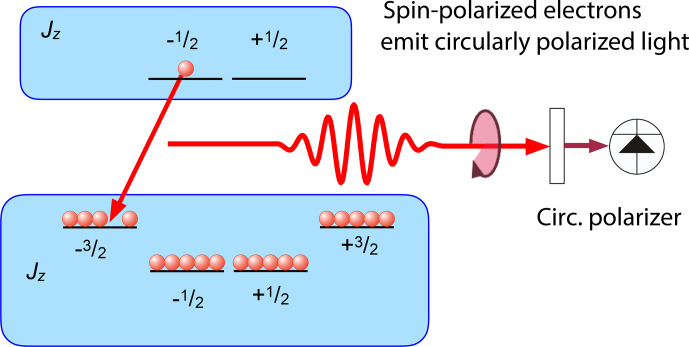
Photoluminescence (PL) from a semiconductor quantum well when the charge carriers are partly spin-polarized. For the case shown here, the emission perpendicular to the well is circularly polarized.

This effect can be used for detecting NMR transitions, as demonstrated in the 1970s and 1980s [Bibr bib1.bibx28] in GaAs. Figure [Fig Ch1.F24] shows the basic principle of optical detection of NMR through the Hanle effect. The red curve represents the Hanle curve of a semiconductor system, whose nuclear field is of the order of 0.65 T. The magnetic field is scanned upwards and at the same time an RF field is applied to the sample. At some 0.65 T, the resonance condition for one of the nuclear spin isotopes in the sample is fulfilled and the RF irradiation saturates the nuclear spins. Accordingly, the nuclear field drops to a much lower value and the PL polarization drops to the value determined by the green curve, which represents the Hanle curve for a total nuclear field of only 0.2 T.

**Figure 24 Ch1.F24:**
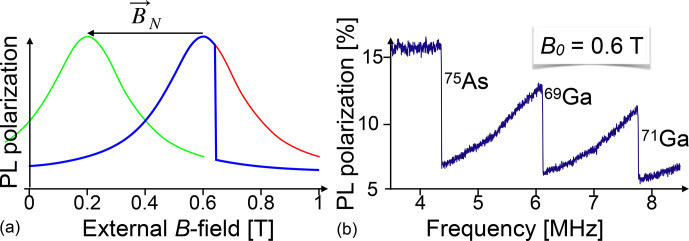
Principle of detection of NMR through the Hanle effect **(a)** and experimental NMR spectrum of a GaAs quantum well **(b)**. PL: photoluminescence.

The right-hand part of the figure shows an experimental spectrum of GaAs, where the external magnetic field is kept constant at 0.6 T, while an RF is applied whose frequency increases from 3.5 to 8.5 MHz. Whenever the frequency reaches the resonance frequency of one of the nuclear spin species present in the sample, it saturates the corresponding spin system, which leads to a reduction of the spin polarization, the corresponding component of the nuclear field, and therefore of the effective magnetic field. This is observed as a sudden drop of the PL polarization at the positions where the resonance occurs for the three isotopes ${}^{69}\mathrm{Ga}$ , ${}^{71}\mathrm{Ga}$ , and ${}^{75}\mathrm{As}$ . Such experiments can target e.g. individual quantum wells in a semiconductor heterostructure [Bibr bib1.bibx31]. They offer a number of applications, such as the measurement of the spin density at different sites within the unit cell from measurements of the Knight shift [Bibr bib1.bibx59].

Similarly to conventional NMR, spectra can be observed in CW mode, i.e. by observing the luminescence and scanning the RF in a constant magnetic field [Bibr bib1.bibx80], or in a time-resolved mode, by recording the freely precessing nuclear spin coherence that causes a modulation of the optical polarization [Bibr bib1.bibx29]. In this case, the nuclear field follows the Larmor precession of the nuclear spins as determined by Eq. ([Disp-formula Ch1.E3]) and the polarization signal maps the Larmor precession. If the nuclear field is weak, the time-dependent polarization signal corresponds directly to the FID signal known from inductively detected NMR. If the nuclear field is strong enough, the transfer function from the spin polarization to the observed signal is nonlinear.

Figure [Fig Ch1.F25] shows, as an example, the Fourier transform of such an FID signal from ${}^{75}\mathrm{As}$ . The signals were obtained with a nuclear field of approximately 1 T, and two effects of the nonlinear response can be observed: the lineshape is distorted (with negative wings) and the spectrum contains not only the central transition and the two quadrupole satellites, but also additional resonance lines at $\omega_{L}\pm 2\omega_{Q}$ , which result from mixing the centre band with the two sidebands by the nonlinear detector response. The simulated spectrum does not take quadrupolar broadening into account, which results in the larger width of the satellites in the experimental spectrum.

**Figure 25 Ch1.F25:**
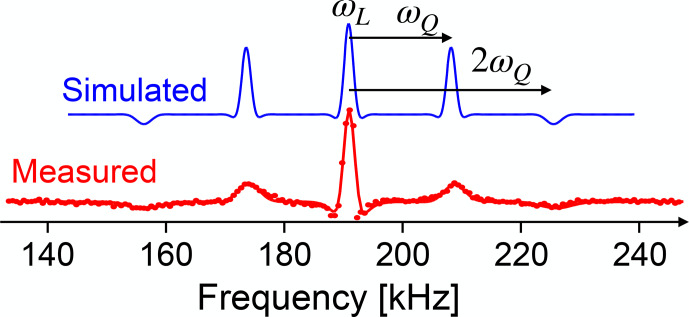
Spectrum of ${}^{75}\mathrm{As}$ obtained by Fourier transformation of the FID signal measured via the Hanle effect after pulsed excitation.

### 5.5 Other approaches to detection of NMR

The experiments discussed in Sect. [Sec Ch1.S5.SS4] use the effect of the nuclear spins on the polarization of the electron spin and thus on the polarization of the photoluminescence to detect the nuclear spin. This indirect detection scheme can be used in many similar experiments, such as optically detected ENDOR [Bibr bib1.bibx57]. In this experiment, optically detected EPR was performed by measuring the change in the total intensity of the photoluminescence when the microwave radiation was turned on [Bibr bib1.bibx37]. This signal was then further modulated by applying an additional RF field resonant with the nuclear spin transitions of nuclei that are coupled to the electron spin of the defect centre. This experiment allowed a precise measurement of the hyperfine interaction between a donor electron and both Ga isotopes, which allowed a tentative assignment of the electron to an interstitial Ga.

Apart from these PL measurements, it is also possible to measure with transmitted [Bibr bib1.bibx101] or reflected [Bibr bib1.bibx93] light. In both cases, the spin polarization in the sample affects the complex index of refraction of the medium, which results in optical circular birefringence and optical circular dichroism, as discussed in Sect. [Sec Ch1.S2.SS4.SSS1]. If the light is off-resonant with respect to the optical transition, the effect is mostly through dispersion and is then known as the Faraday effect.

### 5.6 Quantum films and quantum dots

Technological developments in semiconductor science and technology often rely on so-called quantum-confined heterostructures, where the composition of the material varies over distances of a few nanometres. Ideally, these modifications use lattice-matched material systems, such as GaAs/AlAs, whose lattice constants differ by only $\approx 10^{-3}$ . Accordingly, changing the composition does not significantly affect the structure, but changes the electronic properties. This allows one e.g. to create effective potentials for charge carriers, an important prerequisite for many applications, such as lasers.

Studying these structures by the methods of magnetic resonance would be highly desirable, e.g. to assess the quality of the material and small variations of structural and electronic properties. Since structures with lateral dimensions of a few nanometres contain only a small number of spins, such studies cannot be performed by magnetic resonance with direct detection. However, the large increase in sensitivity offered by optical techniques makes this relatively straightforward. Apart from providing sufficient sensitivity, the additional degrees of freedom of the optical part of the experiment also allow one to selectively excite and detect only signal contributions arising from spins in a selected nanostructure.

Figure [Fig Ch1.F26] shows how the wavelength of the photons can be used to select a specific part of the sample – in this case the 19 nm quantum well. In this example, the sample contains five different quantum wells with different thicknesses. Under non-resonant excitation, all quantum wells generate photons whose wavelengths are characteristic for the thickness of the corresponding quantum wells. In the shown example, the photons were separated in a monochromator, and only the signal from the photons originating in the 19 nm quantum well was analysed. The resulting NMR spectrum (recorded with the CW technique and shown as the bottom trace) shows clear steps when the applied RF field (5 MHz) matched the transition frequency of one of the three isotopes contained in GaAs. The resonance <2 T occurs at half of the field for the ${}^{7}5\mathrm{As}$ transition and belongs to the double quantum transition of this isotope.

**Figure 26 Ch1.F26:**
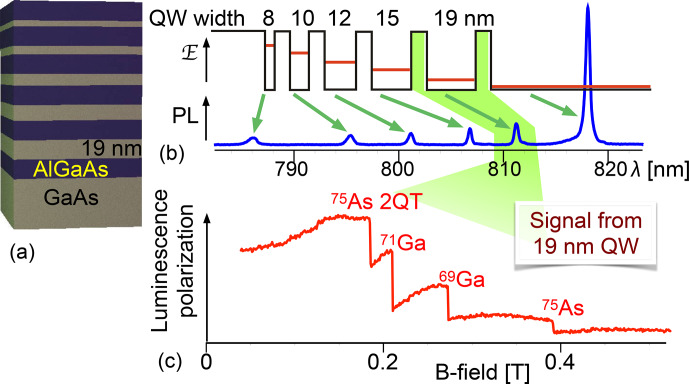
Optical detection of NMR in a single quantum well of a sample with five different quantum wells. **(a)** Structure of the sample, where GaAs quantum wells of different thicknesses are separated by AlGaAs barriers. **(b)** PL spectrum, with assignment of the individual resonance lines to specific quantum wells. Only light from the 19 nm quantum well is detected. **(c)** NMR spectrum of the 19 nm quantum well detected by scanning the magnetic field while applying an RF field with a frequency of 5 MHz.

### 5.7 Quadrupole interactions in semiconductors

The combination of optical pumping with optical detection results in very high sensitivity, which allows one to record spectra with excellent signal to noise even from nanometre-sized structures such as quantum wells. At the same time, the optical excitation and detection allow one to distinguish the signal from these small structures from the much larger signals that the bulk material generates. One interesting application is to study the distortions that are generated at small scales by nanostructures like quantum wells or quantum dots, but also on larger scales by the effect of electric fields or mechanical strain.

Figure [Fig Ch1.F27] shows the basic principle for the example of a nuclear spin I=3/2 , which corresponds to all three isotopes of Ga and As contained in GaAs. In an ideal GaAs crystal, the cubic site symmetry at the Ga and As sites ensures that there is no electric field gradient (EFG), and the energy differences between the four spin states are identical, as they are split only by the Zeeman interaction. However, strain or electric fields, either external ones or fields generated by space charges in the material, can break the symmetry. In those cases, the EFG becomes non-zero and the quadrupole interaction lifts the degeneracy of the magnetic dipole transitions, as shown in the right-hand part of Fig. [Fig Ch1.F27].

**Figure 27 Ch1.F27:**
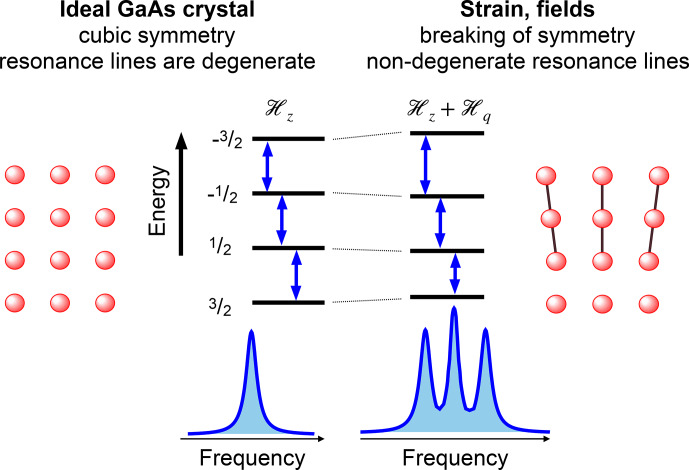
Spectra of I=3/2 spins in ideal and distorted cubic crystals.

Figure [Fig Ch1.F28] shows an example of an NMR signal that was obtained by scanning the RF, while the sample was subject to a constant magnetic field of 0.86 T. The clearly distinguishable steps in the change in the optical signal represent the three allowed transitions in a spin- 3/2 system, as shown schematically in the right-hand part. The two satellite transitions are broader than the central transition, which is not affected by first-order quadrupole interaction.

**Figure 28 Ch1.F28:**
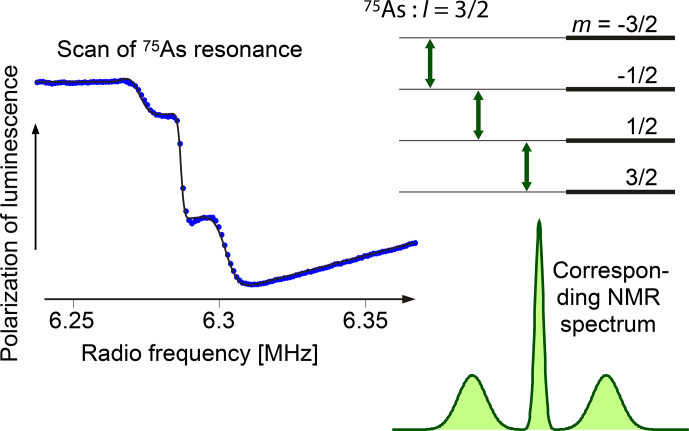
CW NMR spectrum measured by scanning the RF in a constant field of 0.86 T.

Since all three isotopes of GaAs have spin I=3/2 , they all show the same type of quadrupole splitting, although to different degrees. Figure [Fig Ch1.F29] compares the quadrupole splitting of all three isotopes, measured at the same location of the sample. ${}^{75}\mathrm{As}$ shows the largest splitting, while the splitting of the two Ga isotopes is about a factor of 5 smaller. In parallel to the larger quadrupole interaction, the As spectrum shows stronger broadening of the satellite lines, in agreement with expectations for inhomogeneous strain fields.

**Figure 29 Ch1.F29:**
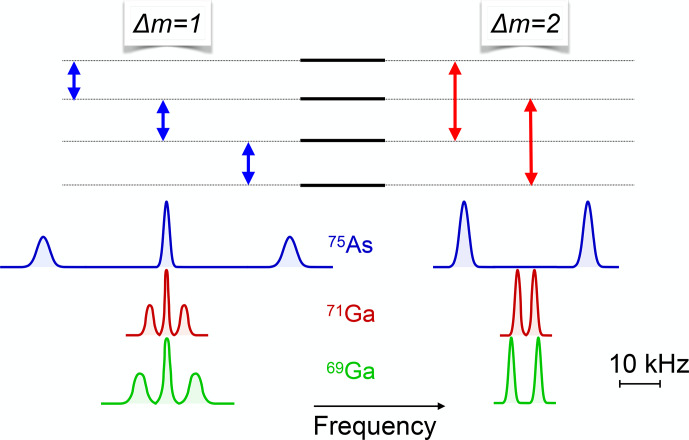
Quadrupole-split spectra of all three isotopes in the same EFG.

The high sensitivity of optical techniques as well as the possibility of using optical excitation to select specific parts of a sample make such experiments highly useful for studying so-called quantum-confined structures. Figure [Fig Ch1.F30] shows the measured quadrupole splittings in a sample containing five quantum wells with different thicknesses. The variation of the quadrupole splitting with depth can be explained [Bibr bib1.bibx31] through the charge distribution in a crystal when two different materials are in contact with each other. This is known as the Schottky effect. Similar experiments have also been performed on different materials like Si, Ge [Bibr bib1.bibx36], and InSb [Bibr bib1.bibx47].

**Figure 30 Ch1.F30:**
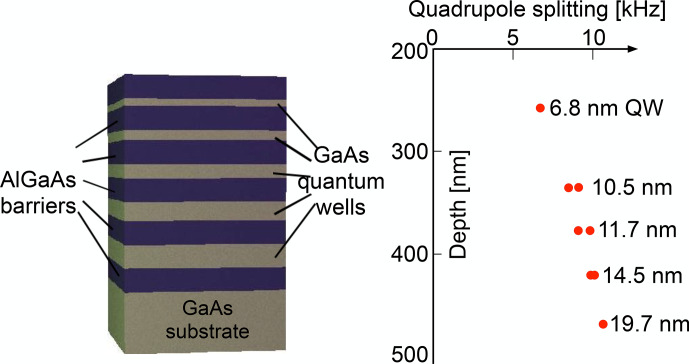
Variation of the quadrupole splitting with the distance from the sample surface.

### 5.8 All-optical excitation and detection of NMR

Most experiments discussed here rely on classical radio-frequency fields to drive the nuclear spin transitions. However, it is also possible to perform experiments purely optically, with no RF irradiation but relying instead on the driving force generated by the optically excited charge carriers [Bibr bib1.bibx30]. While this interaction is generally quite weak, it can be resonantly enhanced by modulating the amplitude or the polarization of the laser field with a frequency near a transition frequency. Both types of modulation act on the nuclear spin via the hyperfine interaction, but the amplitude modulation additionally modulates the charge distribution and therefore the electric field gradient at the nuclear spins. This can be used e.g. to directly drive double quantum transitions, as shown in Fig. [Fig Ch1.F31]. In this case, the modulation frequency has to satisfy the condition $\omega_{\mathrm{mod}}=2\;\omega_{L}$ , where $\omega_{L}$ is the Larmor frequency of the targeted spin. This is fundamentally different from the case of “normal” double quantum excitation with radio-frequency fields, where the condition is $\omega_{rf}=\omega_{L}$ . The experimental data (dots), which correspond to the polarization of the PL, are compared to a simulation (curve), which was calculated for the NMR spectrum shown in the inserts of Fig. [Fig Ch1.F31].

**Figure 31 Ch1.F31:**
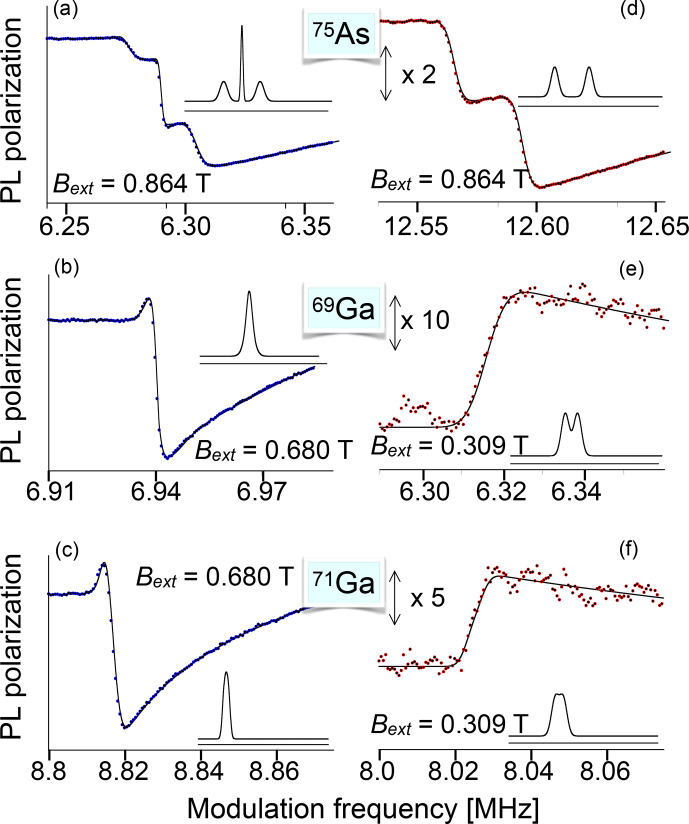
Spectra of single- and double-quantum transitions in GaAs, measured by modulated optical excitation. **(a, b, c)** The polarization of the laser beam was modulated for **(d, e, f)** the amplitude. It therefore excites directly the Δm=±2 transitions.

## 6 Molecular solids

Conventional magnetic resonance has been applied most successfully to molecular systems. These systems can also be investigated with optical methods. Aromatic organic molecules are particularly suitable because their chromophores can support strong optical transitions.

**Figure 32 Ch1.F32:**
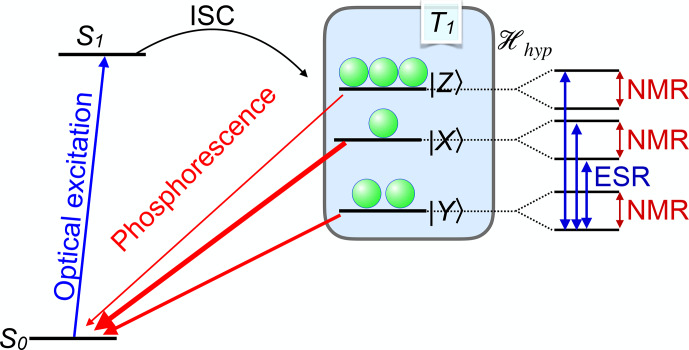
Optical excitation and detection of ESR and NMR transitions in a molecule with a triplet state.

Experiments with these systems typically use optical excitation from the ground state to an excited singlet state with a pulsed UV laser, as shown in Fig. [Fig Ch1.F32]. From the excited singlet state, ISC can populate a nearby triplet state. The ISC populates the different levels of the triplet manifold unequally. In addition, the states of the triplet manifold have in general different lifetimes. As a result, the three different sublevels can have very different populations, as shown schematically in Fig. [Fig Ch1.F32]. The polarization and intensity of the phosphorescence originating from these states depends on the population of the individual states and allows an indirect measurement of the population differences.

By applying microwave fields to the system, it is possible to induce transitions between the different triplet states and thereby change the rate of emitted photons. Accordingly, the transitions can be observed in the photoluminescence. If an appropriate filtering procedure is implemented, most of the collected photons originate from molecules with a high spin polarization. This makes it possible to detect magnetic resonance of individual molecules, such as pentacene in p-terphenyl hosts [Bibr bib1.bibx56]. Initial experiments detected transitions between electron spin states, but soon afterward, nuclear spin transitions in single molecules could also be measured [Bibr bib1.bibx110].

As shown in the right-hand part of Fig. [Fig Ch1.F32], the nuclear spin leads to an additional splitting. The non-thermal populations of the triplet states also lead to nuclear spin polarization, which can survive the return to the ground state. The transfer of polarization from the electron to the nuclei occurs spontaneously through the hyperfine interaction, but can also be induced by microwave irradiation, as in DNP. Adding a radio-frequency field and scanning it over the relevant frequency range then results in optically detected ENDOR or ODENDOR [Bibr bib1.bibx24]. Alternatively, the enhanced nuclear spin polarization can be used by conventional NMR of the ground state.

## 7 Conclusion and outlook

Conventional magnetic resonance uses static and alternating magnetic fields for the study of ensembles of electronic and nuclear spins. This review covers an extension where optical fields are used as an additional tool. These optical fields are usually derived from a laser or they represent luminescence emitted by the sample. The main motivation for such experiments is, in most cases, the increased information content of such double resonance experiments or the increase in sensitivity, which allows sometimes experiments with single spins. In other cases, the correlation between the optical and magnetic resonance degrees of freedom allow one to focus on specific parts of a sample or identify units that cannot be uniquely identified from either modality alone.

While most of the basic principles required for this work have been known for a long time, the actual implementations only became possible through progress in different fields. Major examples are the introduction of tuneable and narrowband laser sources, modulators for light, and sensitive detectors, which are now limited only by quantum mechanics. Applying these new technologies to physical systems was often pioneered by people working in fields like optics (classical or quantum) and molecular or solid-state physics. Conversely, the results of such studies often provide highly valuable information for those fields. Excellent examples of these types of benefits can be found in the fields of semiconductor and surface physics. Currently, the field is still evolving rapidly, and it appears highly probable that new types of applications will be developed for the foreseeable future.

## Data Availability

Data are not publicly available. For further information please contact the corresponding author.
